# Controllable Engineering and Functionalizing of Nanoparticles for Targeting Specific Proteins towards Biomedical Applications

**DOI:** 10.1002/advs.202101713

**Published:** 2021-11-01

**Authors:** Zhanchen Guo, Rongrong Xing, Menghuan Zhao, Ying Li, Haifeng Lu, Zhen Liu

**Affiliations:** ^1^ State Key Laboratory of Analytical Chemistry for Life Science School of Chemistry and Chemical Engineering Nanjing University Nanjing 210023 China

**Keywords:** controllable engineering, epitope, molecular imprinting, nanoparticles, surface functionalization

## Abstract

Nanoparticles have been widely used in important biomedical applications such as imaging, drug delivery, and disease therapy, in which targeting toward specific proteins is often essential. However, current targeting strategies mainly rely on surface modification with bioligands, which not only often fail to provide desired properties but also remain challenging. Here an unprecedented approach is reported, called reverse microemulsion‐confined epitope‐oriented surface imprinting and cladding (ROSIC), for facile, versatile, and controllable engineering coreless and core/shell nanoparticles with tunable monodispersed size as well as specific targeting capability toward proteins and peptides. Via engineering coreless imprinted and cladded silica nanoparticles, the effectiveness and superiority over conventional imprinting of the proposed approach are first verified. The prepared nanoparticles exhibit both high specificity and high affinity. Using quantum dots, superparamagnetic nanoparticles, silver nanoparticles, and upconverting nanoparticles as a representative set of core substrates, a variety of imprinted and cladded single‐core/shell nanoparticles are then successfully prepared. Finally, using imprinted and cladded fluorescent nanoparticles as probes, in vitro targeted imaging of triple‐negative breast cancer (TNBC) cells and in vivo targeted imaging of TNBC‐bearing mice are achieved. This approach opens a new avenue to engineering of nanoparticles for targeting specific proteins, holding great prospects in biomedical applications.

## Graphical abstract

A reverse microemulsion‐confined surface imprinting and cladding approach is developed for the controllable engineering of nanoparticles. It allows for facile, versatile and controllable engineering coreless and core/shell nanoparticles with tunable monodispersed size and targeting capability towards specific proteins and peptides. In vitro and in vivo targeted imaging using nanoparticles prepared by this approach are achieved, showing great prospects in biomedical applications.

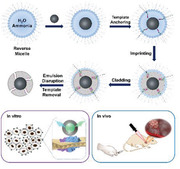

## 1 Introduction

The physical and chemical properties of substances are size‐dependent and significantly enhanced when the size is reduced to nanoscale due to quantum confinement effects. This feature makes nanomaterials highly appealing for various important biomedical applications. For instance, gold and silver nanoparticles, which exhibit unique size‐ and shape‐dependent optoelectronic properties, have found wide applications in biosensing and bioassay. ^[^ ^1^, ^2^, ^3^ ^]^ Superparamagnetic nanoparticles (SPMNPs) have shown great potential in magnetic resonance imaging (MRI), hyperthermia, and biosensing. ^[^ ^4^, ^5^, ^6^ ^]^ Semiconductor nanocrystals or quantum dots (QDs) have become a new class of fluorescent probes for cellular imaging. ^[^ ^7^, ^8^, ^9^ ^]^ Recently, upconverting nanoparticles (UCNPs) have emerged as an appealing candidate for the application of near‐infrared (NIR) light in nanomedicine. ^[^ ^10^, ^11^, ^12^ ^]^ However, the biomedical applications of nanoparticles are often hampered by poor targeting capability. The recognition of nanoparticles toward targets mainly relies on the functionalization of the surface with biological ligands such as antibodies, lectins, and aptamers, which face some inevitable problems. On one hand, due to the distinct differences between these biomolecules and nanomaterials in structure and stability, the properties conferred by such surface functionalization are often nonideal. Particularly, due to nonspecific adsorption, the formation of protein coronas on ligand‐conjugated nanoparticles has been often reported, ^[^ ^13^, ^14^, ^15^ ^]^ which largely degrades the specificity. On the other hand, many kinds of advanced nanoparticles, synthesized in oil phase for controllable size and properties (such as oil‐phase synthetic silver nanoparticles (AgNPs)^[^ ^16^, ^17^, ^18^ ^]^ and UCNPs^[^ ^19^, ^20^, ^21^ ^]^), are often difficult to be functionalized. Their surface functionalization with property transfer from hydrophobic to hydrophilic still remains highly challenging. To this end, an ideal solution should be to directly construct a functional thin layer with specific targeting capability on the surface of substrate nanomaterials via rational design and controllable material synthesis.

As an important methodology to create synthetic mimics of antibodies for the recognition of chemical and biological species, molecular imprinting^[^ ^22^, ^23^, ^24^, ^25^ ^]^ may be an optimal solution to above issue. As compared with antibodies, molecularly imprinted polymers (MIPs) exhibit several merits, including ease in preparation, low cost, chemical, and storage stability. Besides, MIPs can be easily integrated with other functional materials, such as plasmonic nanomaterials, QDs, and so on, to gain dual and even multiple functions to enable various new applications. Due to these merits, MIPs have found applications in many important areas, such as affinity separation, ^[^ ^26^, ^27^ ^]^ disease diagnosis, ^[^ ^28^, ^29^ ^]^ drug delivery, ^[^ ^30^, ^31^ ^]^ targeted bioimaging, ^[^ ^32^, ^33^ ^]^ and cancer therapy. ^[^ ^34^, ^35^ ^]^ However, current imprinting approaches are predominantly noncontrollable so that the core number, particle size, and shape of functionalized nanoparticles often vary dramatically. Although the boronic acid chemistry‐based molecular imprinting strategies^[^ ^36^, ^37^ ^]^ we have developed in recent years have allowed for controllable synthesis of several common substrate‐based imprinted nanomaterials, there are still great gaps between advanced nanomaterials and biomedical applications. Thus, it remains highly desirable to develop facile and versatile approaches for the controllable engineering and functionalization of advanced nanoparticles with specific targeting capability.

Herein, we report a facile, versatile and controllable approach called reverse microemulsion‐confined epitope‐oriented surface imprinting and cladding (ROSIC) for engineering coreless and core/shell nanoparticles with single core, desired size as well as specific targeting capability towards proteins and peptides. Different from the previously reported reverse microemulsion‐based imprinting strategies^[^ ^38^, ^39^ ^]^ using water‐soluble acrylamide monomers for imprinting, taking advantage of silylating monomers, our ROSIC approach allowed for controllable functionalizing of various nanoparticles to gain superior targeting capability. The hydrolyzed silylating monomers during the ROSIC processing participate in the ligand exchange of nanoparticles and facilitates the incorporation of the nanoparticles into the reverse micelles, which is a key step of the reverse‐microemulsion‐based functionalization. More importantly, this approach evolved from our newly developed strategy termed molecular imprinting and cladding (MIC)^[^ ^40^ ^]^ that can effectively overcome to the bottle‐neck problem in conventional molecular imprinting that MIPs prepared under optimized conditions suffer from a compromise between the best affinity and the best specificity. Thus, the cladded MIPs (cMIPs) prepared by ROSIC could provide the best specificity and highest affinity simultaneously. Using QDs, SPMNPs, AgNPs and UCNPs as a representative set of core substrates, we achieved their targeting surface functionalization and successfully prepared a variety of size controllable dual‐functional single‐core @cMIP NPs. To demonstrate the potential of prepared core/shell cMIP NPs in biomedical applications, we prepared single QD‐cored cMIP (QD@cMIP) NPs against two typical cancer biomarkers including human epidermal growth factor receptor‐2 (HER2) and transmembrane glycoprotein non‐metastatic gene B (GPNMB) and achieved differentiation of triple‐negative breast cancer (TNBC) cells over other cell lines via fluorescence imaging. The practical application value was further demonstrated by in vivo targeted imaging of TNBC‐bearing mice using GPNMB‐specific NIR797‐doped cMIP. The ROSIC approach is generally applicable to proteins and peptides. Also, the required templates can be easily obtained through solid‐phase synthesis, obviating the problem with costly and hard‐to‐prepare targets. Moreover, this approach can be adopted to targeting other species of biological significance such as glycans. Therefore, this study opened a new avenue to engineer and functionalization of advanced nanoparticles with targeting capability, holding great prospects in biomedical applications.

## 2 Results and Discussion

**Figure 1:**
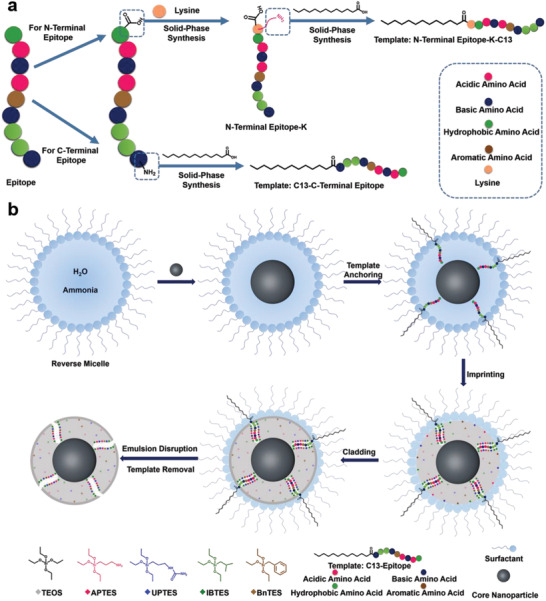
Schematic of the principle and procedure of the ROSIC method. a) template preparation: grafting the epitope with a hydrophobic chain. b) preparation procedure of core/shell cMIP nanoparticles for the recognition of specific proteins and peptides. If no nanocores are added, then the prepared nanoparticles are coreless.

The principle and procedure of the proposed approach are schematically illustrated in **Figure** **1**. To endow nanoparticles to prepare with protein targeting capability, a N‐ or C‐terminal nonapeptide, which is a characteristic fragment of a target protein, was selected as an epitope. The selected epitope was grafted with a hydrophobic fatty acid chain of appropriate length (with a C13 chain in this study), which was used as the imprinting template. C‐terminal epitopes were directly grafted with a fatty acid chain. While for N‐terminal epitopes, a lysine (K) was first introduced to the C‐terminal of the epitopes and then grafted with a fatty acid chain. For the imprinting and cladding, a reverse microemulsion formed with an appropriate surfactant and an oil phase was constructed as a nanoscale reaction cell to confine nanospheres to generate in the aqueous phase. When preparing core/shell cMIPs, nanoparticles to be encapsulated were dispersed into the microemulsion; otherwise, the generated cMIPs were coreless. Due to the presence of a hydrophobic chain on the template, the imprint epitope was anchored at the aqueous/oil interface of the microemulsion, with the imprint epitope protruding in the confined aqueous phase. After the silylating reagents dissolved in the oil phase (cyclohexane in this study) diffused into microemulsion, they hydrolyzed and then polymerized in the aqueous phase. After polymerization for the adequate period (usually 24–48 h), a certain amount of tetraethyl orthosilicate (TEOS) was added to the microemulsion mixture. With TEOS molecules diffusing into the microemulsion, a hydrophilic silica cladding thin‐layer was in situ formed on the surface of the imprinted nanospheres. Finally, the imprinted and cladded nanospheres were washed with an appropriate solution that can disrupt microemulsion and extract the template out of the polymeric shell. This processing generated cMIPs with well‐formed cavities complementary to the template in aspects of shape, size and functionalities but with non‐specific binding sites diminished by the cladding thin layer. Therefore, the prepared core/shell nanoparticles not only maintained the original function of the core nanoparticles but also gained antibody‐comparable affinity and specificity.

In order to obtain high affinity, it is essential to use multiple functional monomers with different functionalities capable of interacting with the target molecules via various interactions. The selection of silylating reagents monomers relies on the property classification of amino acids and functional monomers reported previously. ^[^ ^40^ ^]^ As shown in Figure S1 (Supporting Information), amino acids were classified into five classes: I) acidic, II) basic, III) aromatic, IV) hydrophobic, and V) others. Accordingly, four kinds of functional monomers were selected. Aminopropyltriethoxysilane (APTES), which contains an amino group, was used to interact with class I amino acids through electrostatic attraction as well as class V amino acids via hydrogen bonding. 3‐Ureidopropyl‐triethoxysilane (UPTES), which contains a carbamido moiety, was used to bind with amino acids of class II and class V via mainly hydrogen bonding. Benzyltriethoxysilane (BnTES), which contains a phenyl moiety, was selected to interact with class III amino acids via *π*–*π* stacking interaction. Isobutyltriethoxysilane (IBTES), which contains a hydrophobic moiety, can interact with class IV amino acids via hydrophobic interaction. TEOS does not contain any functional moieties, but it can function as a crosslinker to form a silica skeleton as well as a hydrophilic cladding thin layer to cover nonimprinted area. Since different epitopes contain different numbers of different type of amino acids, the type of the monomers to be used for a specific epitope should be selected according to the kinds of amino acids present in the epitope while the ratio of the monomers should be optimized experimentally.

To demonstrate the general applicability of the proposed method, three important protein disease biomarkers were used as the test targets, including B2M, HER2, and GPNMB. B2M is a biomarker for multiple myeloma while HER2 and GPNMB are two biomarkers for breast cancer. Particularly, GPNMB is a newly discovered biomarker for triple‐negative breast cancer. The amino acid sequences of the epitopes of these targets are shown in Figure S2 (Supporting Information). It should be noted that due to the different sources of GPNMB, two slightly different peptide sequences were used as the epitopes to fabricate cMIPs for recognizing GPNMB. Using the C‐terminal nonapeptide of B2M as the epitope, we first verified that the newly developed MIC strategy^[^ ^40^ ^]^ could be successfully applied in a reverse microemulsion system to develop the ROSIC method, and confirmed the expected binding specificity. In order to better understand the process of the ROSIC method, coreless MIP prepared by conventional reverse microemulsion‐confined epitope‐oriented surface imprinting (ROSI) and coreless cMIP prepared by ROSIC were first prepared.

The composition of the silylating reagents may greatly influence the rate of hydrolysis, which may affect the material formation and ultimately affect the imprinting effect. As the diffusion of the silylating reagents into the microemulsion took time, the reaction time required by the ROSIC method was generally long (typically 24–48 h). Besides, aqueous ammonia acts as both a reactant (H_2_O) and a catalyst (NH_3_) for the hydrolysis of silylating reagents. On one hand, a sufficient amount of ammonia was needed to ensure hydrolysis. On the other hand, too much ammonia would increase the size of the aqueous domain, which might also affect the material formation and ultimately affect the imprinting effect. To facilitate the reaction rate, a certain amount of ammonia was added to the aqueous phase. Therefore, providing that the reaction time was long enough, it was no need to optimize the imprinting time. We optimized the specific ratio of monomers and ratio between total monomers and crosslinking agent (TEOS) in terms of imprinting factor (IF). IF is an essential parameter that reflects the imprinting effect, which is calculated by the ratio of the amount of template molecules captured by the prepared MIPs and cMIPs over that by non‐imprinted polymers (NIPs) and cladded nonimprinted polymers (cNIPs).

**Figure 2:**
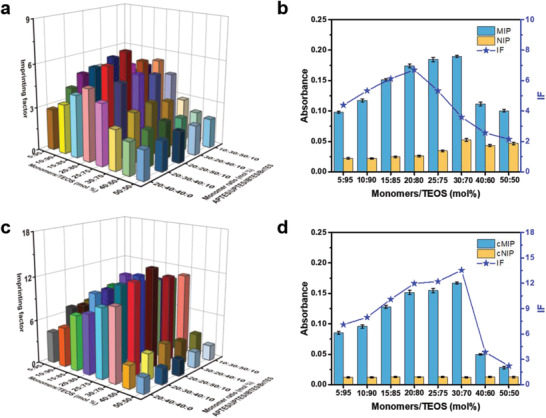
Optimization of imprinting conditions and comparison of imprinting effect. a) Optimization of the imprinting conditions for the preparation of B2M C‐terminal epitope‐imprinted MIPs by non‐cladding approach. b) Comparison of the absorbance for B2M C‐terminal epitope captured by B2M C‐terminal epitope‐imprinted MIPs and NIPs prepared at different total monomers/TEOS ratios under optimal specific monomer ratio (APTES/UPTES/IBTES/BnTES = 20:20:50:10). c) Optimization of the imprinting conditions for the preparation of B2M C‐terminal epitope‐imprinted cMIPs by molecular imprinting and cladding. d) Comparison of the absorbance of the template captured by B2M C‐terminal epitope‐imprinted cMIP and cNIP prepared at different total monomers/TEOS ratios under optimal specific monomer ratio (APTES/UPTES/IBTES/BnTES = 20:20:50:10).

As shown in **Figure** **2** and Figure S3 (Supporting Information), among the specific monomer ratios investigated, the ratio of APTES/UPTES/BnTES/IBTES at 20:20:50:10 yielded the best imprinting effect for both MIP and cMIP, giving an IF value of 6.7 and 13.7 for MIP and cMIP, respectively. This suggests that the best specific monomer ratios for a given imprint were the same for both MIP and cMIP. However, the optimal ratio of total monomers/TEOS was different for MIP and cMIP. The highest IF value for MIP was found at the monomers/TEOS ratio of 20:80, whereas the best ratio of monomers/TEOS for cMIP shifted to 30:70. It can be seen that under all the ratios investigated, the amount of template captured by the NIPs changed apparently, while the amount of template captured by the cNIPs was greatly reduced to a low and constant level, which agrees with the principle of MIC^[^ ^40^ ^]^ and suggests that the cladding process was effective in all cases. Clearly, the improved imprinting effect was due to the fact that the template adsorption capability of nonimprinted surface was greatly suppressed by the cladding thin layer. This indicates the effectiveness of cladding process in ROSIC.

On the other hand, it can be seen that under all specific monomer ratios investigated, the amount of template molecules captured by MIPs and cMIPs increased as increasing the total ratio of the monomer over TEOS within the range from 5:95 to 30:70. However, further increase in the ratio resulted in a dramatic drop in the amount of template captured. To elucidate the mechanism, using SEM and TEM, we characterized the MIP, cMIP, NIP and cNIP nanoparticles prepared at all the monomers/TEOS ratios. The results are shown in Figures S4–S11 (Supporting Information). From Figures S4–S9 (Supporting Information), it can be seen that the prepared nanomaterials showed uniform spherical morphology, with a diameter of about 45 nm. The sizes of nanomaterials prepared by MI and MIC were similar, which indicates that the cladding layer was very thin. We found that when the ratio of monomers/TEOS was 40:60 (Figure S10, Supporting Information), the prepared materials became less uniform, smaller in size, and aggregated. When the ratio was increased to 50:50, this phenomenon became more obvious (Figure S11, Supporting Information). This indicates that the backbone structure and morphology of the prepared materials were mainly determined by the crosslinker TEOS and thereby a high crosslinker/monomer ratio was required.

Using fluorescence‐labeled B2M C‐terminal epitope (FITC‐KIVKWDRDM) as the test compound, the affinity of the materials prepared by ROSI and ROSIC are comparatively investigated. The binding isotherms of B2M C‐terminal epitope‐imprinted MIPs and cMIPs prepared under above optimized conditions were established by plotting the fluorescence intensity for the test compound captured by the materials against the logarithmic concentration of the test compounds. Using corresponding data, the dissociation constants (*K*_d_) of B2M C‐terminal epitope‐imprinted MIPs and cMIPs were measured according to the Scatchard equation. As shown in Figure S12 (Supporting Information), the *K*_d_ values were found to be 1.39 ± 0.39 × 10^–8^ and 1.41 ± 0.39 × 10^–8^ m for MIP and cMIP, respectively, suggesting that high affinity was maintained in ROSIC.

To further investigate target binding capability of the MIPs and cMIPs, we measured the binding affinity and kinetics of the B2M C‐terminal epitope‐imprinted MIPs and cMIPs to the intact protein B2M by biolayer interferometry (BLI). The binding parameters, including dissociation constants (*K*_d_), association rate constants (*k*_on_) and dissociation rate constants (*k*_off_) are listed in Table S1 (Supporting Information). For the MIP and cMIP, the binding curves exhibited typical association/dissociation patterns (Figure S13, Supporting Information), yielding satisfactory correlation coefficients (*R* ^2^ > 0.95). The *K*_d_ values were found to be 7.19 ± 0.08 × 10^–9^ and 1.63 ± 0.34 × 10^–8^ m for the MIP and cMIP, respectively. These data are slightly different from those obtained by the Scatchard equation, which can be assigned to the deviation of different methods. As a positive control, for anti‐B2M monoclonal antibody, the binding curve also exhibited typical association/dissociation pattern (Figure S14, Supporting Information). The *K*_d_ value was found to be 5.06 ± 0.01 × 10^–9^ m. Although the affinity for the cMIP was slightly poorer than that of the monoclonal antibody, it is well acceptable and can be improved by further optimization. Besides, the *k*_off_ values for the MIP, the cMIP and the anti‐B2M monoclonal antibody ranged from 10^−4^ to 10^−3^ s^−1^, which are similar to those for other specific binding, but lower than those for nonspecific binding (10^−2^ to 10^−3^ s^−1^) . ^[^ ^41^, ^42^, ^43^, ^44^ ^]^ These results suggest that the specific affinity of the MIP and cMIP toward the template was due to the well‐fabricated imprinting cavities, rather than nonspecific adsorption by the imprinting coating.

**Figure 3:**
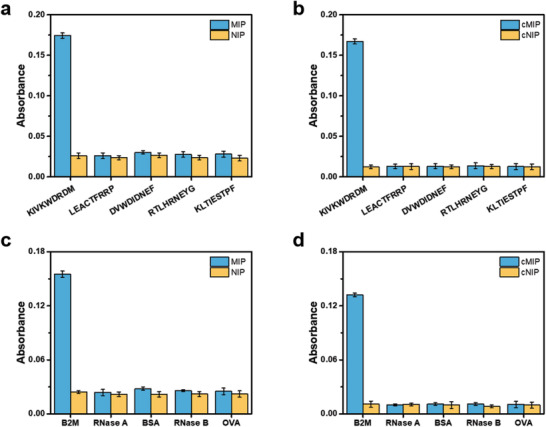
Selectivity test. a) Comparison of the absorbance for test peptide captured by B2M C‐terminal epitope‐imprinted MIP and corresponding NIP prepared under optimal monomer ratios. b) Comparison of the absorbance for test peptide captured by B2M C‐terminal epitope‐imprinted cMIP and corresponding cNIP prepared under optimal monomer ratios. c) Comparison of the absorbance for test protein captured by B2M C‐terminal epitope‐imprinted MIP and corresponding NIP prepared under optimal monomer ratios. d) Comparison of the absorbance for test protein captured by B2M C‐terminal epitope‐imprinted cMIP and corresponding cNIP prepared under optimal monomer ratios.

The specificity of the above prepared MIP and cMIP was comparatively investigated at the peptide level and the protein level. As shown in **Figure** **3**, the MIP showed apparently poor specificity, yielding cross‐reactivity ≤ 16.9% towards the interfering peptides and cross‐reactivity ≤ 19.1% toward the interfering proteins. As a comparison, the cMIP exhibited much improved specificity, giving cross‐reactivity ≤ 8.3% at the peptide level and cross‐reactivity ≤ 8.1% at the protein level. Thus, the expected specificity enhancement by the cladding processing was experimentally confirmed.

**Figure 4:**
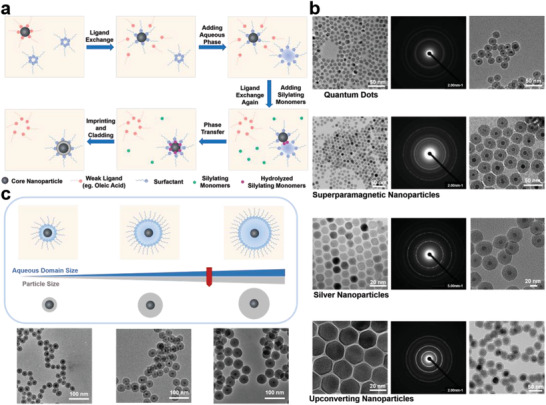
Controllable engineering of core/shell cMIP nanoparticles. a) Functionalization mechanism of pre‐synthesized nanoparticles stabilized with organic ligands by ROSIC. b) TEM images (left), selected area electron diffraction pattern (middle) of CdSe/ZnS quantum dots (QDs), superparamagnetic nanoparticles (SPMNPs), silver nanoparticles (AgNPs) and upconverting nanoparticles (UCNPs) and TEM images of QD@cMIP, Ag@cMIP, MNP@cMIP and UCNP@cMIP (right). c) Illustration and TEM images showing size controllable preparation of QD@cMIP.

By virtue of the unique nanoconfinement effect of reverse microemulsion, we further explored the possibility of the ROSIC approach for controllable engineering of core/cMIP shell nanoparticles. As shown in **Figure** **4**a, the process included five steps: 1) ligand exchange, 2) addition of aqueous phase, 3) ligand exchange again and adding silylating monomers, 4) phase transfer, and 5) imprinting and cladding. The key step of the reverse‐microemulsion‐based functionalization is to introduce nanoparticles into the nanometer‐sized droplets. One simple way is in situ synthesis of nanoparticles and then direct functionalization. However, most nanoparticles need to be presynthesized to obtain particular physical and chemical properties, which can also be functionalized by reverse microemulsion method. The mechanism for the functionalization of nanoparticles stabilized with hydrophilic ligands is straightforward. Due to the hydrophilicity, such nanoparticles can enter the aqueous phase directly. Recently, the silica‐based functionalization mechanism of presynthesized nanoparticles stabilized with organic ligands by reverse microemulsion method has been well elucidated by many researches. ^[^ ^45^, ^46^, ^47^, ^48^ ^]^ Usually, organic ligands, like alkyl amino chains or alkyl acid chains, on the surface of nanoparticles are labile, can be easily replaced by both hydrolyzed silylating monomers and surfactant molecules, which facilitates the incorporation of the nanoparticles into the reverse micelles (Figure 4a).

In this work, we successfully achieved controllable functionalization of various dual‐functional size‐tunable core/shell nanoparticles by ROSIC. As shown in Figure 4b, the prepared QD@cMIP, Ag@cMIP, SPMNP@cMIP, and UCNP@cMIP exhibited uniform and single‐cored morphology, with a uniform diameter. This confirms that the ROSIC process truly achieved precisely controllable engineering functionalization of nanoparticles.

The functional shell of different thicknesses has its own specific function, and its control is very important for different biological applications. It has been reported that the thickness of silica shells could be tuned by changing the amount of silylating monomers like TEOS, but the tuning is very limited and free‐core silica or multicore silica constantly appeared because of the heterogeneous or homogeneous nucleation of SiO_2_. The use of multiple silylating monomers in this work could also make its controllability more challenging. Previous work^[^ ^49^ ^]^ has found that the SiO_2_ shell thickness basically remains constant as the TEOS content increases up to a certain extent, which is due to confinement of aqueous domain size on the overall size of core/shell nanoparticles. This is also the reason to ensure the effectiveness of our ROSIC approach. Inspired by this, it is a suitable way to control the thickness of the overall size of core/shell nanoparticles by controlling the size of the aqueous phase (Figure 4c). The size control of the aqueous phase can be achieved by simply adjusting the composition of microemulsion, like turning the ratio of surfactant to water or change the type of surfactant. In this work, single‐cored and size‐controllable QD@cMIP NPs with size of 30, 40, and 50 nm were successfully prepared by the ROSIC method (Figure 4c).

TNBC refers to a subtype of breast cancer that does not express three major therapeutic targets including estrogen receptor (ER), progesterone receptor (PR), and HER2. So far, effective treatment of TNBC still remains lacking and challenging. GPNMB is a type I transmembrane glycoprotein and expressed in the tumor stroma of 64% of human breast tumors and in the tumor epithelium of an additional 10% of tumors. ^[^ ^50^ ^]^ Recent studies^[^ ^50^, ^51^, ^52^ ^]^ have identified high GPNMB expression in TNBC and suggested GPNMB as a potential biomarker for clinical research. In this work, using QD520 (with emission maximum at 520 nm) and QD620 (with emission maximum at 620 nm) as fluorescent nanocores, GPNMB‐specific QD520@cMIP and HER2‐specific QD620@cMIP were prepared by the proposed ROSIC method for targeting TNBC cells (GPNMB+) and HER2+ breast cancer cells, respectively.

We first optimized the imprinting conditions for the preparation of anti‐GPNMB QD520@cMIP. According to above established knowledge, the ratio of monomers/TEOS was fixed at 30:70. As shown in Figure S15a (Supporting Information), the best imprinting was obtained when the ratio of APTES/UPTES/IBTES/BnTES was 10:30:50:10, giving a high IF value (12.3). Because a single QD was introduced into the cMIP, we further investigated the imprinting effect within a higher monomers/TEOS ratio range (from 30:70 to 50:50) at the optimal specific monomer ratio to check if the previous conclusion was still valid. From Figure S15b (Supporting Information), we found that as increasing the monomers/TEOS, the IF value decreased too. This is in agreement with above knowledge of the requirement of high TEOS/monomers ratio for good material structure. Thus, whether or not the cMIP contains a nanocore, the ratio of monomers/TEOS should be fixed at 30:70. The specificity of anti‐GPNMB QD520@cMIP was investigated. The cross‐reactivity was less than 8.9% at the peptide level and less than 9.3% at the protein level (Figure S15c,d, Supporting Information).

Keeping the ratio of monomers/TEOS at 30:70, we further optimized the imprinting conditions for the preparation of anti‐HER2 QD620@cMIP. Since the structure of N‐terminus of HER2 contains no class III amino acids, BnTES was not used for the preparation of QD620@cMIP. As shown in Figure S16 (Supporting Information), the best monomer ratio was found at APTES/UPTES/IBTES of 10:30:60, giving an IF value of 11.8. The cross‐reactivity was found to be less than 9.0% at the peptide level and less than 9.7% at the protein level.

**Figure 5:**
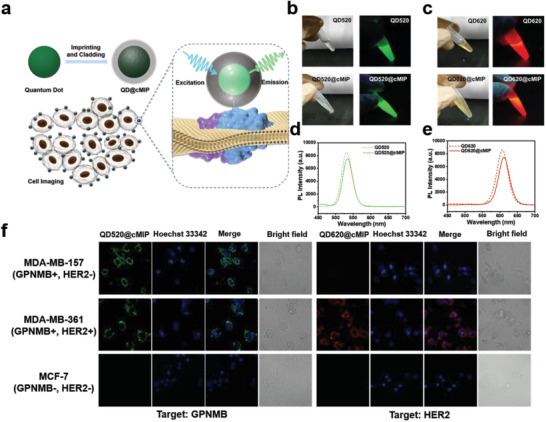
Fluorescence imaging of different human breast cancer cells. a) Illustration of fluorescence imaging for targeting different human breast cancer cells. b) Photos of QD520 before and after molecular imprinting and cladding. c) Photos of QD620 before and after molecular imprinting and cladding. d) Fluorescence spectra of QD520 before and after molecular imprinting and cladding. e) Fluorescence spectra of QD620 before and after molecular imprinting and cladding. f) Confocal fluorescence imaging for targeting different human breast cancer cells by GPNMB‐specific QD520@cMIP and HER2‐specific QD620@cMIP. Blue: nuclei stained by Hoechst 33342.

The prepared nanomaterials were characterized by TEM and EDS elementary mapping. As shown in Figure S17 (Supporting Information), the prepared QD520@cMIP and QD620@cMIP exhibited uniform and single‐cored morphology, with a uniform diameter of about 27 nm. It was found that the MIC process did not significantly affect the fluorescence properties of quantum dots (**Figure** **5**b–e).

To investigate the target recognition and fluorescence imaging capability of the QD520@cMIP and QD620@cMIP, QD520@cMIP, QD520@cNIP, QD620@cMIP, and QD620@cNIP were incubated with human TNBC cells MDA‐MB‐157 (GPNMB+, HER2‐), human breast cancer cells MDA‐MB‐361 (GPNMB+, HER2+) and MCF‐7 cells (GPNMB‐, HER2‐). The expression GPNMB and HER2 proteins of the cells used was first evaluated by Western blot (Figure S18, Supporting Information). As shown in Figure 5, GPNMB N‐terminal epitope‐imprinted QD520@cMIP showed very strong fluorescence intensity towards MDA‐MB‐157 and MDA‐MB‐361 cells, but no obvious fluorescence signals towards MCF‐7 cells. HER2 N‐terminal epitope‐imprinted QD620@cMIP showed very strong fluorescence intensity to MDA‐MB‐361 cells, but no obvious fluorescence signals to MDA‐MB‐157 and MCF‐7 cells. As a comparison, QD520@cNIP and QD620@cNIP had no fluorescence signals to the above three cells (Figure S19, Supporting Information). The quantitative flow cytometry analysis shown in Figure S20 (Supporting Information) verified the target recognition capability of the QD520@cMIP and QD620@cMIP. Besides, immunofluorescence imaging and the corresponding quantitative flow cytometry analysis of anti‐GPNMB antibody and anti‐HER2 antibody were carried out as the positive control groups (Figures S21 and S22, Supporting Information). These results indicate that the prepared QD@cMIP NPs exhibited antibody‐comparable targeting performance in fluorescence imaging as well as quantitative flow cytometry, showing the great potential of QD@cMIP NPs in biomedical applications.

The practical application value of the ROSIC approach in biomedical applications was further evaluated. The in vitro stabilities of MIPs and cMIPs were first investigated. As shown in Figure S23 (Supporting Information), the prepared MIP and cMIP were negatively charged in water, which means that they could be well distributed in water and physiological solution. Then, the stabilities of MIP and cMIP in phosphate‐buffered saline (PBS) and fetal bovine serum (FBS) solutions were further investigated. For better observations, the coreless B2M C‐terminal epitope‐imprinted MIP and cMIP were used here. As shown in Figure S24 (Supporting Information), for MIP dispersed in PBS, it began to precipitate on day 3 and almost completely precipitated on day 5. As a comparison, when the cMIP was dispersed in PBS, it still remained well dispersed even on day 7. Both the MIP and cMIP could be dispersed well in FBS and they did not precipitate even on day 7. Clearly, the cladding process was highly favorable to the stability of the cMIP in real‐world environment. This indicates the importance of cladding process in the ROSIC approach for biomedical applications.

The cytotoxicity of MIP and cMIP were also investigated by the 3‐(4,5‐dimethylthiazol‐2‐yl‐)‐2,5‐diphenylterazolium bromide (MTT) assay. And anti‐GPNMB QD520@MIP and anti‐GPNMB QD520@cMIP were used. As shown in Figure S25 (Supporting Information), when the concentration was lower than 400 µg mL^−1^, both anti‐GPNMB QD520@MIP and anti‐GPNMB QD520@cMIP were nearly nontoxic to both normal cells and cancer cells. This means that our ROSIC method can effectively functionalize the nanomaterials and enhance its biocompatibility, showing an extremely high potential for biomedical applications.

**Figure 6:**
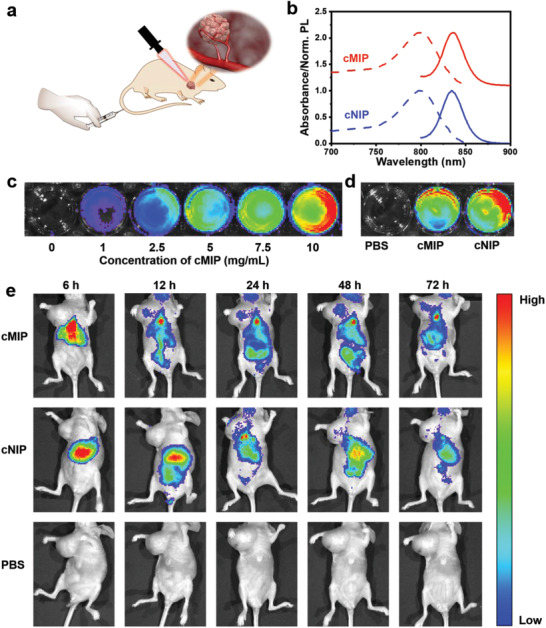
In vivo fluorescence imaging of MDA‐MB‐157 tumor after intravenous injection of different nanomaterials. a) Illustration of in vivo fluorescence imaging of targeting MDA‐MB‐157 tumor after intravenous injection of different nanomaterials. b) Evolution of the optical absorption (dashed lines) and photoluminescence (PL) (solid lines) spectra of NIR797‐doped cMIP and NIR797‐doped cNIP. c) Fluorescence intensity mapping of NIR797‐doped cMIP at different concentrations. d) Fluorescence intensity mapping of PBS, NIR797‐doped cMIP, and NIR797‐doped cNIP. e) In vivo fluorescence imaging of MDA‐MB‐157 tumor after intravenous injection of GPNMB‐specific NIR797‐doped cMIP, NIR797‐doped cNIP and PBS.

Finally, the in vivo targeting capability of cMIP was investigated by preparing GPNMB‐specific NIR797‐doped cMIP and NIR797‐doped cNIP. The preparation of NIR797‐doped cMIP was straightforward. NIR797‐doped silica dots were in situ synthesized first and then direct functionalized by the ROSIC approach. The prepared nanoparticles could be excited and emitted in the near‐infrared I region so that it could be used for in vivo fluorescence imaging (**Figure** **6**b–d). The in vivo targeting capability of GPNMB‐specific NIR797‐doped cMIP was investigated by fluorescence imaging of the biodistribution of the cMIP in MDA‐MB‐157 tumor‐bearing male nude mice. Figure 6e shows the biodistribution of the GPNMB‐specific NIR797‐doped cMIP, NIR797‐doped cNIP and PBS after intravenous injection for 6, 12, 24, 48, and 72 h, respectively. For mice injected with GPNMB‐specific NIR797‐doped cMIP, the injected materials started to appear at the tumor site at 12 h and the fluorescence intensity became stronger as the time after the intravenous injection increased. For the mice injected with NIR797‐doped cNIP, the nanomaterial was mainly located at the liver and only a limited amount was accumulated at the tumor site and lasted for a short period. The short‐term and limited residence of cNIP at the tumor site was attributed to the enhanced permeability and retention (EPR) effect. For mice injected with PBS, no fluorescence signal was observed all the time, which is reasonable since no fluorescent substance was injected. These results indicate that the cMIP could specifically recognize MDA‐MB‐157 tumor while the cNIP failed to do so.

## 3 Conclusion

In this study, we have developed a versatile imprinting method called ROSIC for controllable engineering and conferring single nanoparticles with specific targeting capability towards proteins and peptides. Coreless and single‐core/shell nanoparticles with tunable size and special response including fluorescent, superparamagnetic, plasmonic and upconverting response have been successfully prepared using this method. Practical application values of the prepared molecularly imprinted and cladded nanoparticles have been well demonstrated via in vitro and in vivo targeted imaging of TNBC. The ROSIC method is applicable to any protein and peptide targets providing that their terminal epitopes are accessible. The templates required can be easily prepared via solid‐phase synthesis. The approach can be also adopted to engineering coreless and core/shell nanoparticles for targeting other species such as glycans. Due to its versatility, controllability and effectiveness, we foresee the great respective of the developed method in biomedical applications.

## 4 Experimental Section

### General Procedure of the ROSIC Method

The preparation procedure was composed of four steps: 1) reverse microemulsion formation and template anchoring; 2) reverse microemulsion‐confined interface epitope imprinting, 3) cladding, and 4) microemulsion disruption and template removal.

### Microemulsion Formation and Template Anchoring

The microemulsion consisted of 1.77 g of Triton X‐100, 1.6 mL of n‐hexanol, 6.5 mL of cyclohexane, 480 µL of water, and 100 µL of ammonium hydroxide. It was stirred at 700 rpm at 25 °C to form a clear and transparent solution. During this procedure, the following steps were performed: a monomer solution was first prepared by adding 100 µL of different molar ratios of APTES, UPTES, IBTES, and BnTES in 1 mL of cyclohexane and vortexed for 1 min, and the obtained solution is designated as S1. Then, a cross‐linker solution was prepared by adding 100 µL of TEOS in 1 mL of cyclohexane and vortexed for 1 min, and the resulting solution is designated as S2. Finally, 1 mL of solution for imprinting was prepared by mixing an appropriate volume of S1 of different molar ratio of monomers and S2 and vortexed for 1 min, which is designated as S3. Fresh solutions for imprinting were prepared prior to the imprinting.

### Reverse Microemulsion‐Confined Interface Epitope Imprinting

The solution was stirred for 30 min in a 25 mL eggplant‐shaped flask with a Teflon‐coated, egg‐shaped magnetic stir bar with the length of 1.5 cm to get a clear and transparent solution. Then, 1 mg of C13 fatty acid‐grafted epitope was added to the microemulsion solution and continuously stirred for another 30 min. After that, 1 mL of S3 was dropwise added carefully. The mixture was stirred at 700 rpm at 25 °C for 24 h.

### Cladding

After imprinting, 100 µL of TEOS was added dropwise to the mixture, followed by stirring at 700 rpm for another 24 h at 25 °C. The prepared epitope‐imprinted cMIP was released from the microemulsion by adding acetone, followed by centrifugation at 4000 rpm for 30 min to separate the cMIP from the reaction mixture. The obtained cMIP was washed with anhydrous ethanol and water five times each.

### Microemulsion Disruption and Template Removal

The obtained epitope‐imprinted cMIP was dispersed into 5 mL of ACN:H_2_O:HAc = 50:49:1 (v/v) and shaken for 20 min at room temperature. The above elution process was repeated three times. After removing the C13‐grafted epitope template, the prepared epitope‐imprinted cMIP was collected by centrifugation at 4000 rpm for 30 min. The obtained cMIP was washed with water and anhydrous ethanol three times each and then freeze‐dried in a vacuum overnight. The product was stored at 4 °C for future use.

For epitope‐imprinted MIPs prepared with noncladding imprinting approach, the preparation process was the same except that the prepared nanoparticles were precipitated and collected directly after reverse microemulsion‐confined interface epitope imprinting and no cladding process was performed. For cladded nonimprinted polymers (cNIPs) and nonimprinted polymers (NIPs), the preparation process was the same except that no epitope templates were added.

### Preparation of NaYF_4_: Yb, Tm Upconversion Nanoparticles

The UCNPs were synthesized using a modified method. ^[^ ^19^ ^]^ First, 0.747 mL of 1 m YCl_3_, 0.25 mL of 1 m YbCl_3_ and 0.1 mL of 0.03 m TmCl_3_ aqueous solutions were pipetted into a 100 mL three‐necked round‐bottom flask and heated at 110 °C to evaporate off the water. The stirring speed was maintained at 350 rpm throughout the synthesis. 6 mL of oleic acid and 15 mL of 1‐octadecene were added into the flask, the mixture was heated up to 150 °C and kept it for 30 min to form a clear and homogeneous solution. Then, the heating mantle was removed and allowed the reaction mixture to cool down to 60 °C. 5 mL of methanol containing 0.1 g of NaOH and 0.148 g of NH_4_F was added into the reaction mixture dropwise and heated to 110 °C for 20 min to evaporate off the methanol and residual moisture. Last, the flask was sealed with a glass stopper and connected it to the dual manifold line through a condenser. The reaction mixture was kept under vacuum for 10 min. The two‐way stopcock of the manifold line was switched to interchange the atmosphere in the flask between vacuum and argon for three cycles (1 min for each cycle). Next, the flask was filled with argon atmosphere and raised the temperature to 300 °C at a heating rate of 10 °C min^‐1^. The temperature was maintained for 1 h. After that, the heating mantle was removed and allowed the reaction mixture to cool down to room temperature. The reaction mixture was transferred into a 50 mL centrifuge tube, the flask was rinsed with acetone for three times and then transferred it into the centrifuge tube. The mixture was topped up to 40 mL with acetone. The mixture was vortexed for 1 min and centrifuged the tube at 6700 g for 10 min at room temperature. The supernatant was discarded and dissolved the pellet in 20 mL of cyclohexane by vortexing, and then centrifuged the tube at 1000 g for 5 min at room temperature. The supernatant containing UCN core was stored in a 20 mL glass scintillation vial for future use (designated as **UCN solution**).

### Synthesis of Iron‐Oxide Nanocrystals with a Particle Size of 12 nm Synthesis of Iron‐Oleate Precursor

In a typical synthesis, ^[^ ^53^ ^]^ iron chloride (10.8 g, 40 mmol) was first dissolved in a mixture of 80 mL ethanol and 60 mL Nanopure water in a three‐neck flask (500 mL). Afterward, sodium oleate (36.5 g 120 mmol) was quickly added to the iron chloride solution along with 140 mL hexane. The resulting solution was allowed to stir until the sodium oleate was completely dissolved. Next, the reaction solution was heated to ≈58 °C for a 4 h reflux. When the reaction was finished and cooled to room temperature, the upper organic layer containing the iron oleate was washed three times with 30 mL of Nanopure water in a 250 mL separatory funnel. Excess hexane was then evaporated using a rotovap. The resulting iron oleate was transferred into a 100 mL round bottom flask and connected to a Schlenk line where it was placed under a vacuum of ≈50 mtorr overnight, and then the iron oleate was well sealed in a glass vial and stored in a desiccator for 2 d of aging.

### Synthesis of Iron‐Oxide Nanocrystals with a Particle Size of 12 nm: Synthesis of Iron‐Oxide Nanocrystals

The iron‐oxide nanocrystals were synthesized using a modified method. ^[^ ^53^, ^54^ ^]^ Iron oleate (4 mmol, 3.6 g) and oleic acid (2.2 mmol, 0.624 g) were added into a three‐neck flask (100 mL) with 1‐octadecene (20 g). The mixture was stirred under argon flow at room temperature. After 10 min, the reaction solution was heated to 320 °C at a heating rate of ≈18 °C min^‐1^. Reaction time was counted from the moment when 320 °C was reached. After 1 h reaction, the reaction solution was quickly cooled to room temperature by blowing air across the reaction flask. The resulting iron‐oxide nanocrystals were precipitated using acetone, and then were purified using three rounds of precipitation/redispersion cycles with acetone and hexane as the solvents. After purification, the product was dispersed in cyclohexane for future use.

### Synthesis of Single‐Crystalline Ag Nanocrystals with a Particle Size of 15 nm

The Ag nanocrystals were synthesized using a modified method. ^[^ ^17^ ^]^ In a typical synthesis, dodecane (20 mL) was placed in a 100 mL three‐neck flask and heated to 170 °C by a digitally controlled heating mantle with vigorous magnetic stirring and Ar flowing. A mixture of 1.0 µL of HCl (0.012 mmol), 0.30 mL of dodecane, and 0.20 mL of oleylamine was injected into the flask. After 10–20 s, a freshly prepared solution of 0.040 g of silver acetate (CH_3_COOAg; 0.240 mol) dissolved in 2.0 mL of oleylamine was quickly injected into the flask. The reaction temperature was maintained at 170°C for synthesis. After 15 min, 2‐hexyldecanoic acid (0.20 mL, 0.68 mmol) was injected into the flask to improve the colloidal stability of the nanocrystals. Subsequently (5 min later), a mixture of acetic acid (40 µL, 0.70 mmol) and an equal amount of oleylamine was injected into the flask. After 90 min, the reaction was stopped by removing the heating mantle and blowing air on the surface of the reaction flask. The resulting nanocrystals were precipitated using acetone, and then were purified using three rounds of precipitation/redispersion cycles with acetone and hexane as the solvents. After purification, the product was dispersed in 20 mL of cyclohexane for future use (designed as **Ag solution**).

### General Procedure for the Preparation of Single‐Cored cMIPs by the ROSIC Method

The preparation procedure was similar as described above except the introduction of various nanoparticle solutions during the ***microemulsion formation*** procedure. The microemulsion was first stirred at 700 rpm for 10 min at 25 °C, and nanoparticle solution was then added and stirred for 30 min before C13‐grafted epitope was added. To prepare QD@cMIP, 700 µL of QD520 solution (3 mg mL^‐1^ in cyclohexane) was added. To prepare Fe_3_O_4_@cMIP, 1 mL of Fe3O4 solution (2 mg mL^‐1^ in cyclohexane) was added. To prepare UCNP@cMIP, 4 mL of as‐prepared UCN solution was added. To prepare Ag@cMIP, 2 mL of as‐prepared Ag solution was added. The other procedures were all the same as above.

## Conflict of Interest

The authors declare no conflict of interest.

## Supporting information

Supporting Information

## Data Availability

Research data are not shared.
