# Supplementary material for: Controllable Engineering and Functionalizing of Nanoparticles for Targeting Specific Proteins towards Biomedical Applications
Source: Adv Sci (Weinh). 2021 Nov 1;8(24):2101713. doi: 10.1002/advs.202101713 (PMC8693047; doi:10.1002/advs.202101713)
Supplement: Supplementary file 1 — Supporting Information [file ADVS-8-2101713-s001.pdf]

© 2021 Wiley-VCH GmbH

ADVANCED SCIENCE
Open Access

Supporting Information

for *Adv. Sci.*, DOI: 10.1002/advs.202101713

Controllable Engineering and Functionalizing of Nanoparticles for Targeting Specific Proteins towards Biomedical applications

*Zhanchen Guo, Rongrong Xing, Menghuan Zhao, Ying Li, Haifeng Lu, and Zhen Liu**

Supporting Information

Controllable Engineering and Functionalizing of Nanoparticles for Targeting Specific Proteins towards Biomedical Applications

*Zhanchen Guo, Rongrong Xing, Menghuan Zhao, Ying Li, Haifeng Lu, and Zhen Liu**

State Key Laboratory of Analytical Chemistry for Life Science, School of Chemistry and Chemical Engineering, Nanjing University, Nanjing 210023, China

E-mail: zhenliu@nju.edu.cn

METHODS

Reagents. β_2-Microglobulin (B2M), ribonuclease A (RNase A), ribonuclease B (RNase B), bovine serum albumin (BSA), ovalbumin (OVA), Triton X-100, yttrium(III) chloride hexahydrate, ytterbium(III) chloride hexahydrate, thulium(III) chloride hexahydrate, oleic acid (technical grade, 90%), 1-octadecene (technical grade, 90%), oleylamine (technical grade, 70%), NIR-797 isothiocyanate (suitable for fluorescence, 70+% (coupling to amines)) and iron chloride ($FeCl_3 \cdot 6H_2O$, 98%) were obtained from Sigma-Aldrich (St. Louis, MO, USA). Silver acetate (99%) and n-dodecane (99+%) were purchased from Alfa-Aesar. Sodium oleate and 2-hexyldecanoic acid (98%) were purchased from Tokyo Chemical Industry. Recombinant protein human epidermal growth factor receptor-2 (HER2), anti-GPNMB antibody, anti-HER2 antibody, FITC-conjugated goat anti-rabbit IgG H&L secondary antibody and Alexa Fluor® 647-conjugated goat anti-rabbit IgG H&L secondary antibody was purchased from Abcam (Shanghai, China). Human protein GPNMB, which has an additional alanine at its N-terminal, was purchased from Biorbyt (Cambridge, UK). Anti-β_2-microglobulin antibody was purchased from Sino Biological (Beijing, China). Unmodified and modified peptides, including KIVKWDRDM, LEACTFRRP, DVWDIDNEF, RTLHRNEYG, KLTIESTPF, KRFHDVLGN, TQVCTGTDM, C13-KIVKWDRDM, AKRFHDVLGNK-C13, TQVCTGTDMK-C13 and FITC-KIVKWDRDM, were customer-ordered from Shanghai Top-Peptide Biotechnology (Shanghai, China) and their purities (HPLC) were above 98%. Aminopropyltriethoxysilane (APTES), 3-ureidopropyl-triethoxysilane (UPTES), benzyltriethoxysilane (BnTES), isobutyltriethoxysilane (IBTES) and tetraethyl orthosilicate (TEOS) were purchased from J&K Scientific (Shanghai, China). Hexane, n-Hexanol and cyclohexane were purchased from Aladdin Reagent (Shanghai, China). Ammonium hydroxide and anhydrous ethanol were purchased from Nanjing Reagent Company (Nanjing, China). Ammonium fluoride, methanol and acetonitrile (ACN) were purchased from Shanghai Macklin Biochemical (Shanghai, China). Hydrochloric acid (HCl) and acetic acid (HAc) were purchased from Sinopharm Chemical

Reagent Co. I.td. Human triple negative breast cancer cell MDA-MB-157 (over expression of GPNMB, low expression of HER2), breast cancer cell MDA-MB-361(over expression of GPNMB and HER2) and MCF-7 (low expression of GPNMB and HER2), male SPF mice (5 weeks, 20-25 g), phosphate-buffered saline for cell culture (1× PBS), parenzyme cell digestion solution (containing 0.25% trypase and 0.02% EDTA), Dulbecco Modified Eagle Medium (DMEM, containing 4.5 mg/ml glucose, 80 U/ml penicillin and 0.08 mg/ml streptomycin), Roswell Park Memorial Institute 1640 medium (RPMI-1640, containing 2.0 mg/mL D-glucose, 0.3 mg/mL glutamine, 2.0 mg/mL $NaHCO_3$, 80 U/mL penicillin and 0.08 mg/mL streptomycin), dimethyl sulfoxide (DMSO) and 96-well plates were purchased from Nanjing KeyGen Biotech. (Nanjing, China). 4',6-Diamidino-2-phenylindole (DAPI) stain solution (ready-to-use), Hoechst 33342 stain solution (ready-to-use) and 4% formaldehyde were purchased from Beijing Solarbio Science & Technology (Beijing, China). Fetal bovine serum (FBS) was purchased from Gibco (Life Technologies, Australia). 3-(4,5-Dimethylthiazol-2-yl)-2,5-diphenyltertrazolium bromide (MTT), tissue or cell total protein extraction kit , BCA protein assay kit, SDS-PAGE gel preparation kit, HRP–conjugated secondary antibodies, molecular weight standard and enhanced chemiluminescence (ECL) kit were purchased from Beyotime Biotechnology (Shanghai, China). Amino-modified quantum dots (QD520 and QD620) dissolved in cyclohexane were purchased from Xingzi New Material Technology Development (Shanghai, China). Culture cells for cell culture and confocal imaging were purchased from NEST Biotechnology (Wuxi, China). All other reagents used were of analytical grade or higher. Water used in all the experiments was purified by a Milli-Q Advantage A10 water purification system (Millipore, Milford, MA, USA). All chemicals were used directly without any further purification unless otherwise stated.

Instruments. Transmission electron microscopic (TEM) characterization was carried out on a JEM-2800 system (JEOL, Tokyo, Japan). Scanning electron microscope (SEM)

characterization was carried out on a JSM-7800F system (JEOL, Tokyo, Japan). Ultraviolent (UV) spectral analysis was performed with a NanoDrop 2000/2000C spectrophotometer (Thermo Fisher, MA, USA). Microplate reader analysis was carried out on a BioTek Synergy Mx microplate reader (Winooski, VT, USA). Bio-layer interferometry (BLI) binding assays were determined on an Octet Red 96 instrument (FroteBio, CA, USA), and APS biosensors and protein A biosensors were used for all the determinations. Excitation-emission spectra was carried on an FLS980 fluorescence spectrometer (Edinburgh Instruments, UK). Cell imaging was carried out on an LSM 710 laser scanning confocal microscopy (Zeiss, Oberkochen, Germany). Flow cytometric analysis was performed on a Beckman Coulter CytoFlex S system (California, USA). Western blot assay was carried out on a Mini-PROTEAN® Tetra Cell System (Bio-Rad, California, USA), and the imaging was performed on a GelDoc-XRTM gel imaging device (Bio-Rad, California, USA). In vivo imaging experiment was performed on a PerkinElmer Caliper IVIS Lumina XR III system (Waltham, Massachusetts, USA).

Optimization of imprinting conditions of B2M-specific cMIP. The specific monomer ratio and ratio between total monomer and TEOS used for the preparation of epitope-imprinted cMIPs were optimized in terms of the obtained imprinting factor (IF). Epitope-imprinted cMIPs and cNIPs (2.0 mg each) prepared at different specific molar ratios of monomers (APTES, UPTES, IBTES, and BnTES) and different total monomers/TEOS ratios were added separately to 200 μL of phosphate buffer (10 mM pH 7.4) containing 0.1 mg/mL of epitope. After incubation at room temperature for 30 min, the nanoparticles were collected by centrifugation at 4,000 rpm for 30 min and rinsed with 200 μL of phosphate buffer (10 mM, pH 7.4) three times. The nanoparticles were re-suspended and eluted in 20 μL of ACN:H_2O:HAc = 50:49:1 (v/v) at room temperature for 10 min on a rotator. Finally, the suspensions were centrifuged at 4,000 rpm for 30 min and the supernatant were collected. The amount of epitope was determined by measuring the UV absorbance of the supernatant at 214 nm. The measurement

was repeated three times. For control experiments, all the procedures were the same as described above except the absence of epitope in the test samples. For optimization of conventional epitope-imprinted polymers, all the procedures were the same as described above except that cMIPs and cNIPs were replaced by MIPs and NIPs prepared under otherwise identical conditions, respectively.

Selectivity test of B2M-specific cMIP. The selectivity of B2M C-terminal epitope-imprinted cMIP at the peptide level was evaluated using the C-terminal epitopes of B2M (KIVKWDRDM), TRF (LEACTFRRP) and TfR (LEACTFRRP), and the N-terminal epitopes of alpha fetoprotein (AFP) (RTLHRNEYG) and carcinoembryonic antigen (CEA) (KLTIESTPF) as test peptides. First, each epitope standard solution (0.1 mg/mL) was separately prepared with phosphate buffer (10 mM, pH 7.4). Then equivalent corresponding cMIP and cNIP (2 mg each) were added to 200 μL of the epitope solutions in 250-μL micro-centrifugal tubes. The tubes were shaken on a rotator at room temperature for 30 min. The nanoparticles were collected by centrifugation at 4,000 rpm for 30 min and rinsed with 200 μL of phosphate buffer (10 mM, pH 7.4) three times. Second, the nanoparticles were re-suspended and eluted in 20 μL of ACN:H_2O:HAc = 50:49:1 (v/v) at room temperature for 10 min on a rotator. Finally, the nanoparticles were precipitated via centrifugation and the supernatant were collected. The amount of epitope bound by the cMIP was determined by measuring the UV absorbance of the supernatant at 214 nm. The measurement was repeated three times. For control experiments, all the procedure was the same as described above except the absence of epitope in the test samples. For the selectivity test of B2M C-terminal epitope-imprinted cMIP in the protein level, all the procedure was the same as described above except that the test peptides used were changed to the proteins B2M, RNase A, BSA, RNase B and OVA.

For the selectivity test of B2M C-terminal epitope-imprinted MIP at the peptide and protein level, all the procedures were the same as described above except that the B2M C-

terminal epitope-imprinted cMIP and cNIP were replaced by B2M C-terminal epitope-imprinted MIPs and NIPs, respectively.

Measurement of adsorption isotherm. A series of standard solutions of fluorescently labeled B2M C-terminal epitope (FITC-KIVKWDRDM) of known concentrations were prepared with phosphate buffer (10 mM, pH 7.4). A volume of 200 μL of the above standard solutions was added to a 96-well plate, and the fluorescence intensity was measured by a microplate reader. Then 2 mg of the B2M C-terminal epitope-imprinted cMIP were separately added to 1 mL of the above standard solutions and shaken at room temperature for 30 min. After the nanoparticles were centrifuged, 200 μL of the supernatant was added to a 96-well plate and their fluorescence intensity was measured by the microplate reader. An adsorption isotherm was established by plotting the difference between the fluorescence intensity of the standard solution before extraction and of the supernatant after extraction using B2M C-terminal epitope-imprinted cMIP against the logarithmic concentration of FITC-KIVKWDRDM. To estimate the binding affinity of B2M C-terminal epitope-cMIP, the amount of FITC-KIVKWDRDM bound by the B2M C-terminal epitope-cMIP was plotted according to the Scatchard equation as given below:

$$\frac{Q_e}{[S]} = \frac{Q_{max}}{K_d} - \frac{Q_e}{K_d}$$

where Q_e, [S], Q_{max} and K_d are the amount of FITC-KIVKWDRDM bound by the B2M C-terminal epitope-cMIP in terms of fluorescence intensity at equilibrium, the free concentration at adsorption equilibrium, the saturated adsorption amount in terms of fluorescence and the dissociation constant, respectively. By plotting Q_e / [S] versus Q_e, K_d and Q_{max} can be calculated from the slope and intercept, respectively.

For the adsorption isotherm of B2M C-terminal epitope-imprinted MIP, all the procedure was the same as described above except that the B2M C-terminal epitope-imprinted cMIP were changed to B2M C-terminal epitope-imprinted MIP.

Dissociation constants (Kd) determination by bio-layer interferometry (BLI). For dissociation constants (Kd) determination of MIP and cMIP to the whole B2M protein, MIPs or cMIPs were immobilization on APS biosensors. The assay procedure included five steps: (1) baseline: sensors immersed in PBS buffer for 60 s, (2) loading, sensors immersed in 2 mg/mL MIP or cMIP solution for 300 s, (3) baseline: sensors immersed in PBST (PBS with 0.1% Tween 20) buffer for 180 s, (4) association: sensors immersed with target solution of different concentrations in PBST buffer for 5 min, and (5) dissociation: sensors immersed in PBST buffer for 5 min. The shake speed was at 1,000 rpm and the solution volumes used were all 200 μL. The response data and affinity parameter were obtained using the ForteBio Data analysis (ver. 12). All the response data obtained were subtracted from the signal for blank controls, which only contained buffer. For the binding assay of anti-B2M antibody, the antibodies were immobilized onto protein A biosensors while the other conditions were the same as above.

Preparation of GPNMB-specific QD520@cMIP. The solution S3 described in ***microemulsion formation*** procedure was used for the imprinting, while slightly different peptide sequences were used as the epitope depending on the targets to test. For selectivity test and the optimization of imprinting conditions, the decapeptide AKRFHDVLGNK, which has an additional alanine at its N-terminal as compared with the native nonapeptide epitope, was used as the epitope. This was due to the fact that the protein GPNMB used in this study remained an additional alanine as its N-terminal. However, the use of additional amino acid in the epitope will not the recognition of the prepared cMIP towards its alanine-deleted analog (KRFHDVLGNK) in selectivity test (Figure S 13). While for the imaging of GPNMB-overexpressed cell lines, the native nonapeptide KRFHDVLGNK was used as the epitope (see Figure S 2 for the structure). For the imprinting, the microemulsion was first stirred at 700 rpm for 10 min at 25 ºC, and 700 μL of QD520 solution (3 mg/mL in cyclohexane) was then added

and stirred for 30 min. 1 mg of C13-grafted epitope was added to the above solution and continued stirring for another 30 min. After that, 1 mL of S3 was added dropwise carefully, and the mixture was allowed to stirred at 700 rpm at 25 ºC for 24 h. Subsequently, 100 μL of TEOS was added dropwise to the mixture, and the mixture was stirred at 700 rpm for another 24 h at 25 ºC. The obtained materials were released from the microemulsion by adding acetone, followed by centrifugation at 4,000 rpm for 30 min and washed with anhydrous ethanol and water five times, respectively.

To remove the C13-grafted epitope, the obtained QD520@cMIP was dispersed into 5 mL of ACN:H_2O:HAc = 50:49:1 (v/v) and shaken for 20 min at room temperature. The above elution process was repeated three times. After removing the C13-grafted epitope template, the prepared GPNMB N-terminal epitope-imprinted QD520@cMIP was collected by centrifugation at 4,000 rpm for 30 min. The collected QD520@cMIP was washed with water and anhydrous ethanol three times each and then freeze-dried in a vacuum overnight.

For the preparation of QD520@cNIP, the process was the same except that no templates were added.

Preparation of HER2-specific QD620@cMIP. The N-terminal epitope of HER2 was used, the preparation process was the same as above except the C13-grafted GPNMB N-terminal epitope and QD520 were changed to C13-grafted HER2 N-terminal epitope and QD620, respectively.

Optimization of imprinting conditions of GPNMB-specific QD520@cMIP. The specific monomer ratios and the total monomers/TEOS ratios used for the preparation of GPNMB N-terminal epitope-imprinted QD520@cMIP were optimized in terms of the obtained IF value. The optimization procedure was the same as describe above, except the materials were changed to GPNMB N-terminal epitope-imprinted QD520@cMIP.

Optimization of imprinting conditions of HER2-specific QD620@cMIP. The specific monomer ratio used for the preparation of HER2 N-terminal epitope-imprinted QD620@cMIP was optimized in terms of the obtained IF value. The optimization procedure was the same as describe above, except the materials were changed to HER2 N-terminal epitope-imprinted QD620@cMIP.

Selectivity test of GPNMB-specific QD520@cMIP and HER2-specific QD620@cMIP. The selectivity test of QD520@cMIP and QD620@cMIP at the peptide and protein levels was carried out using the same procedure described above except that the B2M C-terminal epitope-imprinted cMIP and cNIP were replaced by QD520@cMIP and QD520@cNIP or QD620@cMIP and QD620@cNIP.

In vitro cytotoxicity of GPNMB-specific QD520@MIP and QD520@cMIP. Cell viability was determined by the MTT assay. Briefly, MCF-7 or MCF-10A cells were seeded on 96-well microplates with a density around 10,000 cells per well and allowed to adhere for 24 h prior to the assay. The cells were incubated with different concentrations of GPNMB-specific QD520@MIP or GPNMB-specific QD520@cMIP at 37 °C for 24 h. The zero concentration group was used as control and others were used as test group. And wells without cells were used as background group. Then 50 μL of MTT indicator dye (1 mg/mL in PBS) was added. After incubating for another 4 h at 37°C in the dark, the supernatant was discarded, and 150 μL of DMSO was added to each well. After shaking for 10 min on a shaking table, the optical density of the solution was monitored on the microplate reader. Absorbance was measured at a wavelength of 550 nm. The cell viability was expressed as a percentage of the absorbance of test cells (added with GPNMB-specific QD520@MIP or GPNMB-specific QD520@cMIP) over that of control experiment (without the addition of GPNMB-specific QD520@MIP or

GPNMB-specific QD520@cMIP) (both were deducted by the background absorbance), which can be calculated by the following equation:

$$\text{Cell viability (\%)} = \frac{Abs\ (test) - Abs\ (background)}{Abs\ (control) - Abs\ (background)} \times 100\%$$

Western blot. MCF-7 cells were cultured in the RPMI-1640 medium with 10% FBS for 2-3 days (37 ºC, 5% CO2), while MDA-MB-157 and MDA-MB-361 cells were cultured in the DMEM medium with 10% FBS for 2-3 days (37 ºC, 5% CO2). After that, cell lysis buffer containing protease inhibitor was added on the cells and the cells were lysed on ice for 30 min. Cells were scraped with clean cell scrapes and transferred into centrifugal tubes with a pipette. The lysate mixture was centrifuged in -4 °C at 12,000 rpm for 5 min and the supernatant was cellected for further use. Protein concentration was quantified using BCA protein quantitation assay, and 50 μg of protein from cell lysate was loaded on sodiumdodecyl sulphate-polyacrylamide gel electrophoresis (SDS-PAGE). Polyvinylidene fluoride (PVDF) membrane was used to transfer the gels. After blocking with 5% BSA in TBST (Tris-buffered saline with 0.1% Tween 20), the membrane was incubated with primary antibodied at 4 °C overnight and then secondary antibodies at room temperature for 1 h. Primary antibodies against GPNMB or HER2 were used at 1:1,000 dilution and secondary antibodies were used at 1: 5,000 dilution. Unbound antibodies were washed away with the TBST, then the membrane was incubated with ECL chemiluminescence solution. The Bio-Rad GelDoc-XRTM gel imaging system was employed to expose the membrane and obtain images. β-actin was used to ensure equal loading.

Cell culture and imaging. MCF-7 cells were cultured in the RPMI-1640 medium with 10% FBS for 2-3 days (37 ºC, 5% CO_2), while MDA-MB-157 and MDA-MB-361 cells were cultured in the DMEM medium with 10% FBS for 2-3 days (37 ºC, 5% CO_2). Each cell line was cultured in four batches. The cell culture medium was removed and the cells remained on the cell culture dishes were washed with 1×PBS twice. Then the cells were fixed with 4% formaldehyde for 15

min respectively, followed by incubated with 200 μL of 200 μg/mL GPNMB N-terminal epitope-imprinted QD520@cMIP, QD520@cNIP, HER2 N-terminal epitope-imprinted QD620@cMIP and QD620@cNIP dissolved in 1× PBS for 30 min. Then 1×PBS and free nanoparticles were removed and the remaining cells were rinsed with 1×PBS three times and then stained with 100 μL of Hoechst 33342 for 10 min. After rinsed with 1×PBS twice, the cell culture dishes were supplemented with 1 mL of 1×PBS. The obtained cells were imaged under laser scanning confocal microscopy.

For immunofluorescence, the cells were fixed with 4% formaldehyde for 15 min, followed by incubation with 5% bovine serum albumin in PBS for 60 min to block the nonspecific binding sites. Then the cells were stained with anti-GPNMB antibody or anti-HER2 antibody for 60 min, followed by fluorescent goat anti-rabbit IgG H&L secondary antibody for 60 min. Primary antibodies against GPNMB or HER2 and secondary antibodies were used at 1:1,000 dilution. After that 1×PBS and free antibodies were removed and the remaining cells were rinsed with 1×PBS three times and then stained with 100 μL of DAPI for 10 min. After rinsed with 1×PBS twice, the cell culture dishes were supplemented with 1 mL of 1×PBS. The obtained cells were imaged under laser scanning confocal microscopy.

Flow cytometry. For flow cytometry assay of nanoparticles, MDA-MB-157, MDA-MB-361 and MCF-7 cells were respectively stained with 200 μL of 200 μg/mL GPNMB N-terminal epitope-imprinted QD520@cMIP, QD520@cNIP, HER2 N-terminal epitope-imprinted QD620@cMIP and QD620@cNIP dissolved in 1× PBS for 30 min. Then 1×PBS and free nanoparticles were removed and the remaining cells were rinsed with 1×PBS three times. After that the cells were digested with parenzyme cell digestion solution (containing 0.25% tryptase and 0.02% EDTA) for 2-3 min. The obtained cells were centrifuged at 1,000 rpm for 3 min. After removing the supernatant, the cells were washed with 1×PBS twice and filtrated with 200

mesh sieves. The obtained cell suspensions were injected into cytoanalyzer and the count of cells was set to 10,000.

For flow cytometry assay of antibodies, the cells were harvested and washed first and the total cell number were determined. Then the cells were resuspended to approximately 1-5×10^6 cells/mL in ice cold PBS. And 100 μL of cell suspension was added to each tube, followed by incubation with ice cold 5% bovine serum albumin in PBS for 60 min to block the nonspecific binding sites. After that, the cells were stained with anti-GPNMB antibody or anti-HER2 antibody at 1:500 dilution for 60 min. The cells were washed three times by centrifugation at 400 g for 5 min and resuspended in ice cold PBS. Then the cells were stainied with fluorescent goat anti-rabbit IgG H&L secondary antibody in 5% bovine serum albumin in PBS at 1:1,000 dilution for 60 min in the dark, followed by centrifugation at 400 g for 5 min. After removing the supernatant, the cells were washed with 1×PBS three times and filtrated with 200 mesh sieves. The obtained cell suspensions were injected into cytoanalyzer and the count of cells was set to 10,000.

Preparation of GPNMB-specific NIR797-doped cMIP or cNIP. The amino group of APTES can react with the isothiocyanate of NIR797 to yield a thiourea bridge. Typically, a volume of 5 μL of APTES and 2 mg NIR797 were dissolved in 1 mL of ethanol. After reaction in darkness with vigorous stirring for 12 h, NIR797-derivatized APTES was formed in the solution. The final products were obtained by removing ethanol by rotary evaporation. For the imprinting, the solution S3 described in ***microemulsion formation*** procedure was used for the imprinting, while slightly different peptide sequences were used as the epitope depending on the targets to test. The microemulsion was first stirred at 700 rpm at 25 ºC for 30 min. And above prepared NIR797-derivatized APTES mixed with 10 μL of TEOS was added and stirred for 30 min. Then 1 mg of C13-grafted epitope was added to the above solution and continued stirring for another 30 min. After that, 1 mL of S3 was added dropwise carefully, and the mixture was allowed to

stirred at 700 rpm at 25 ºC for 24 h in the dark. Subsequently, 100 μL of TEOS was added dropwise to the mixture, and the mixture was stirred at 700 rpm at 25 ºC for another 24 h in the dark. The obtained materials were released from the microemulsion by adding acetone, followed by centrifugation at 4,000 rpm for 30 min and washed with anhydrous ethanol and water five times, respectively.

To remove the C13-grafted epitope, the obtained NIR797-doped cMIP was dispersed into 5 mL of ACN:H_2O:HAc = 50:49:1 (v/v) and shaken for 20 min at room temperature. The above elution process was repeated three times. After removing the C13-grafted epitope template, the prepared GPNMB N-terminal epitope-imprinted NIR797-doped cMIP was collected by centrifugation at 4,000 rpm for 30 min. The collected NIR797-doped cMIP was washed with water and anhydrous ethanol three times each and then freeze-dried in a vacuum overnight.

For the preparation of NIR797-doped cNIP, the process was the same except that no templates were added.

In vivo imaging of mice. All the animal experiments were performed in accordance with the ethical guidelines approved by the Animal Care Committee of the Affiliated Drum Tower Hospital of Nanjing University. Male MDA-MB-157 tumor-bearing mice were divided into 3 groups and injected with ifferent materials: 1) GPNMB-specific NIR797-doped cMIP; 2) NIR797-doped cNIP; 3) 1×PBS (control). The injection does was about 5 mg/every mice. The distribution of NPs in mice was imaged using the PerkinElmer in vivo imaging system. The mice were anesthetized with isoflurane and images were taken at different times. All the mice were euthanized after the experiments.

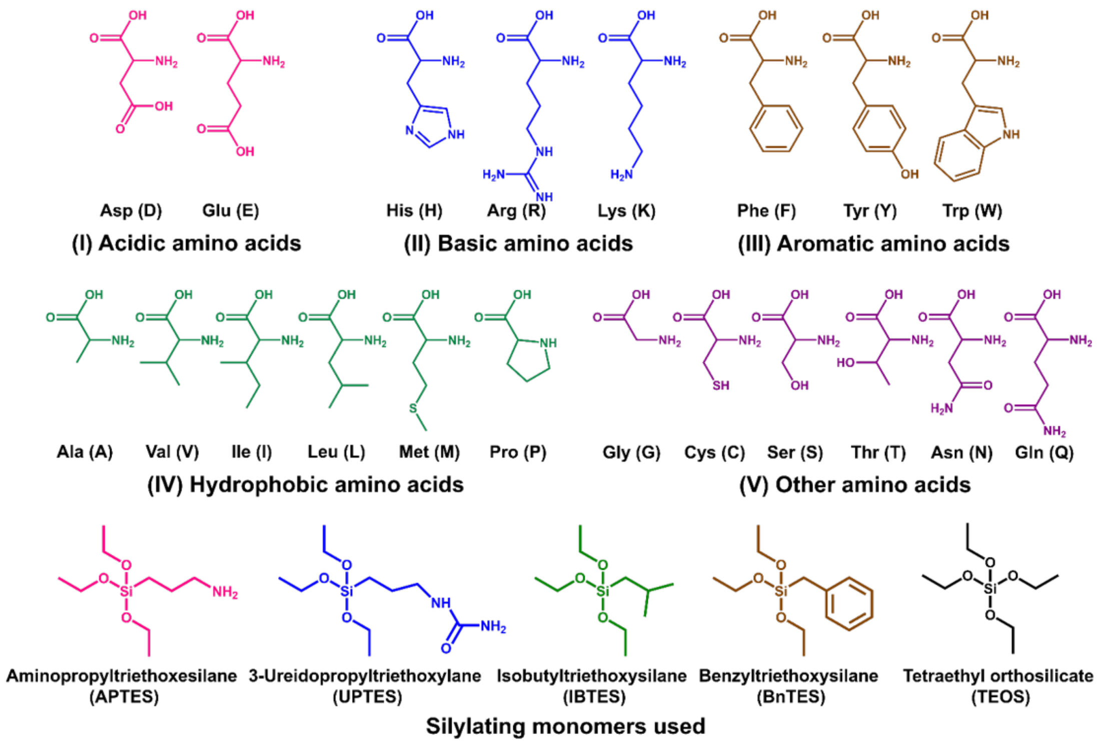

Figure S1. Structures of amino acids and silylating monomers. Amino acids are classified in terms of their properties enabling interactions with silylating monomers (Amino acids and monomers shown in the same color indicate those that can interact with each other).

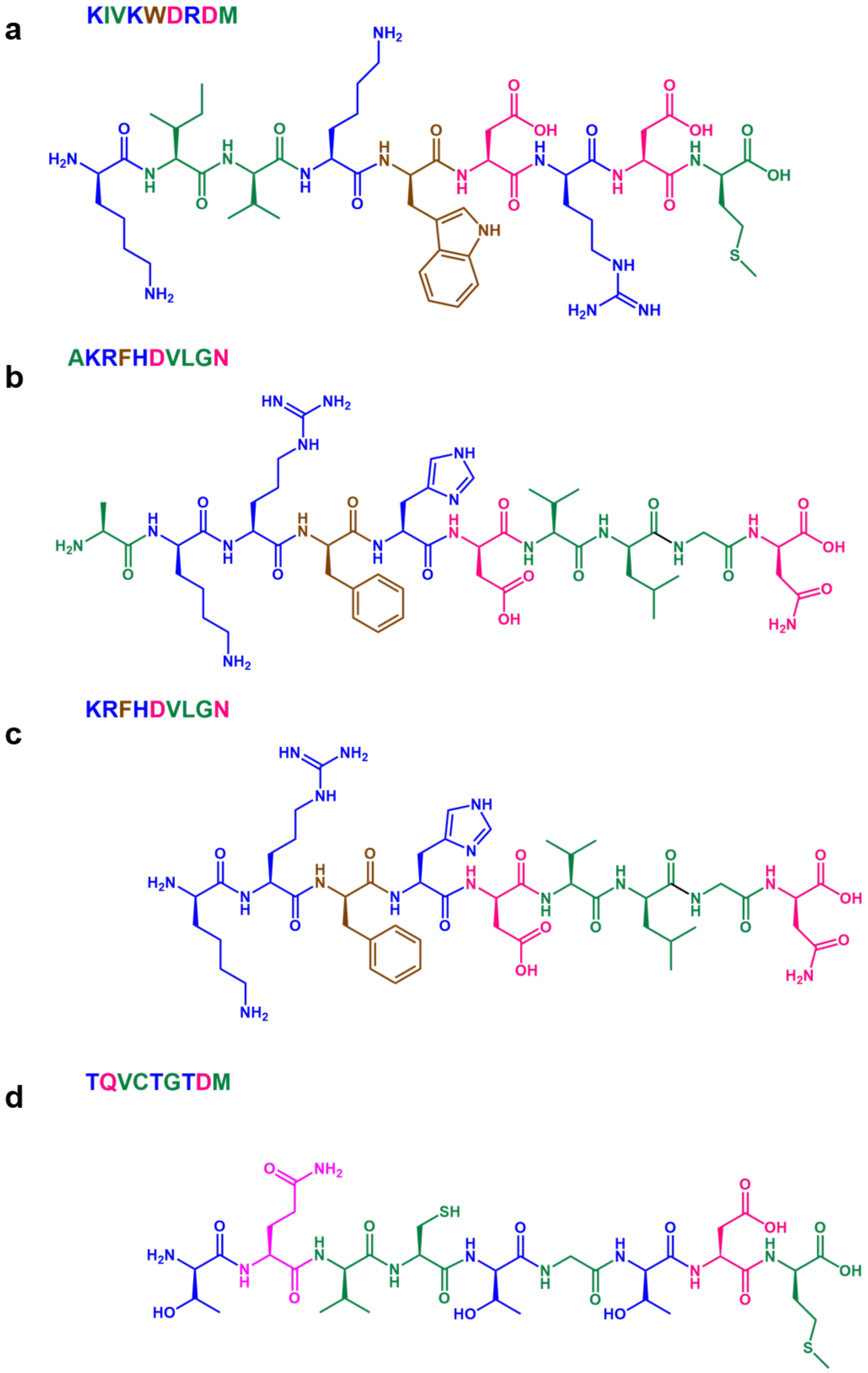

Figure S2. Structures of the epitopes used in this study. a) C-terminal epitope of B2M. b) N-terminal epitope of GPNMB used for specificity test. c) N-terminal epitope of GPNMB used for cell imaging. d) N-terminal epitope of HER2.

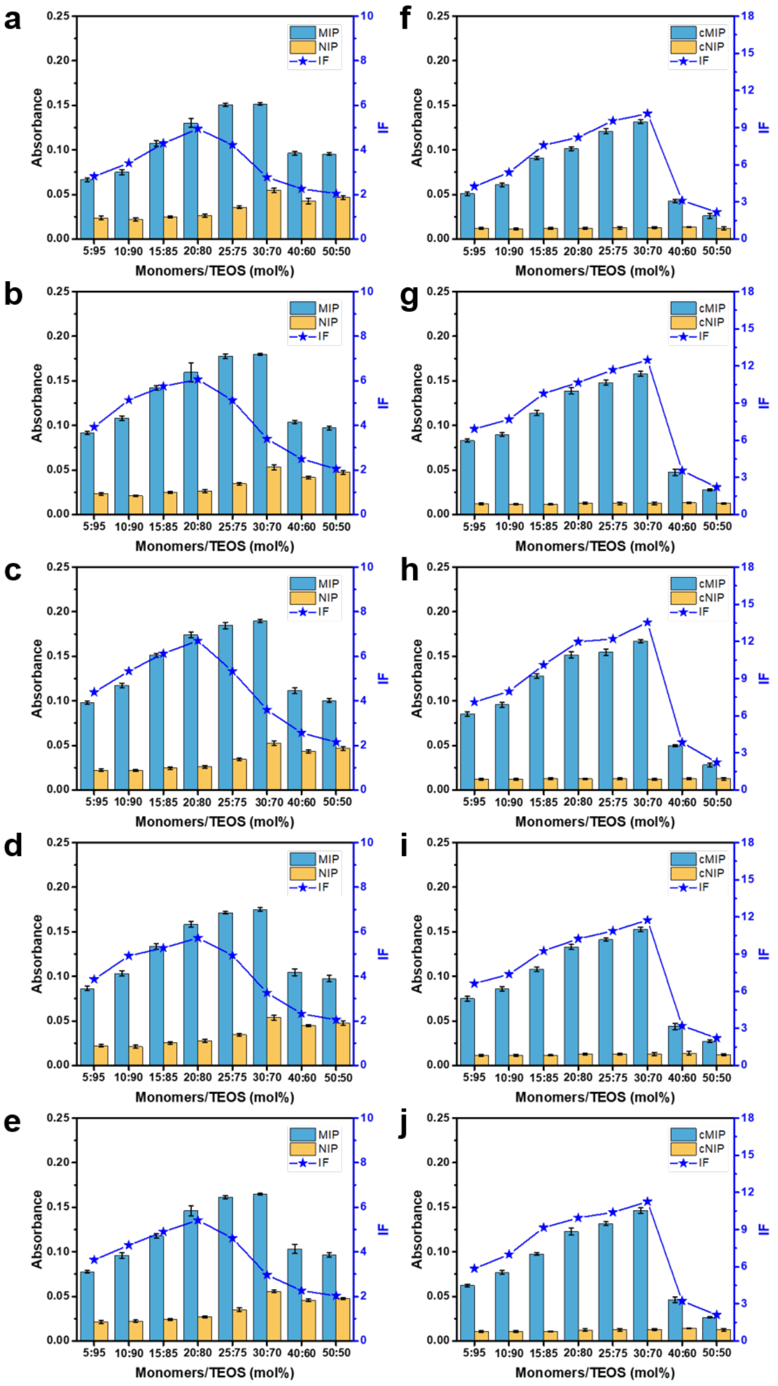

Figure S3. Optimization of imprinting conditions and comparison of imprinting effect. a-e) Comparison of the absorbance of the template captured by B2M C-terminal epitope-imprinted MIPs prepared at different ratio of monomers/TEOS using the ratio of APTES/UPTES/IBTES/BnTES=20:40:40:0 (a), 20:30:40:10 (b), 20:20:50:10 (c), 30:20:40:10 (d), 10:30:50:10 (e). f-j) Comparison of the absorbance of the template captured by B2M C-terminal epitope-imprinted cMIPs prepared at different ratio of monomers/TEOS using the ratio of APTES/UPTES/IBTES/BnTES=20:40:40:0 (f), 20:30:40:10 (g), 20:20:50:10 (h), 30:20:40:10 (i), 10:30:50:10 (j).

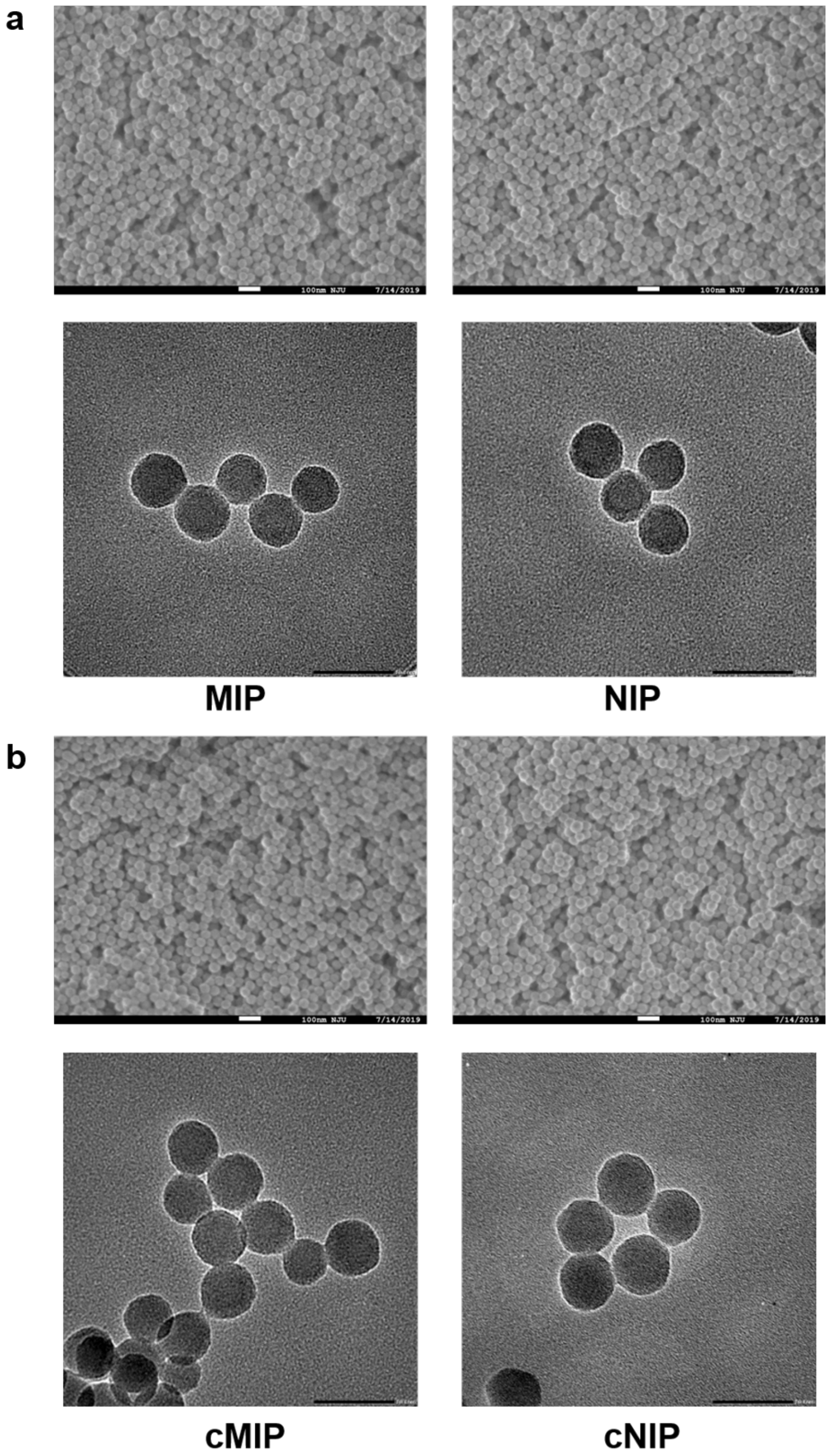

Figure S4. SEM and TEM characterization of nanoparticles prepared at the total monomers/TEOS ratio of 5:95. a) MIP and NIP. b) cMIP and cNIP. The APTES/UPTES/IBTES/BnTES ratio was 20:20:50:10.

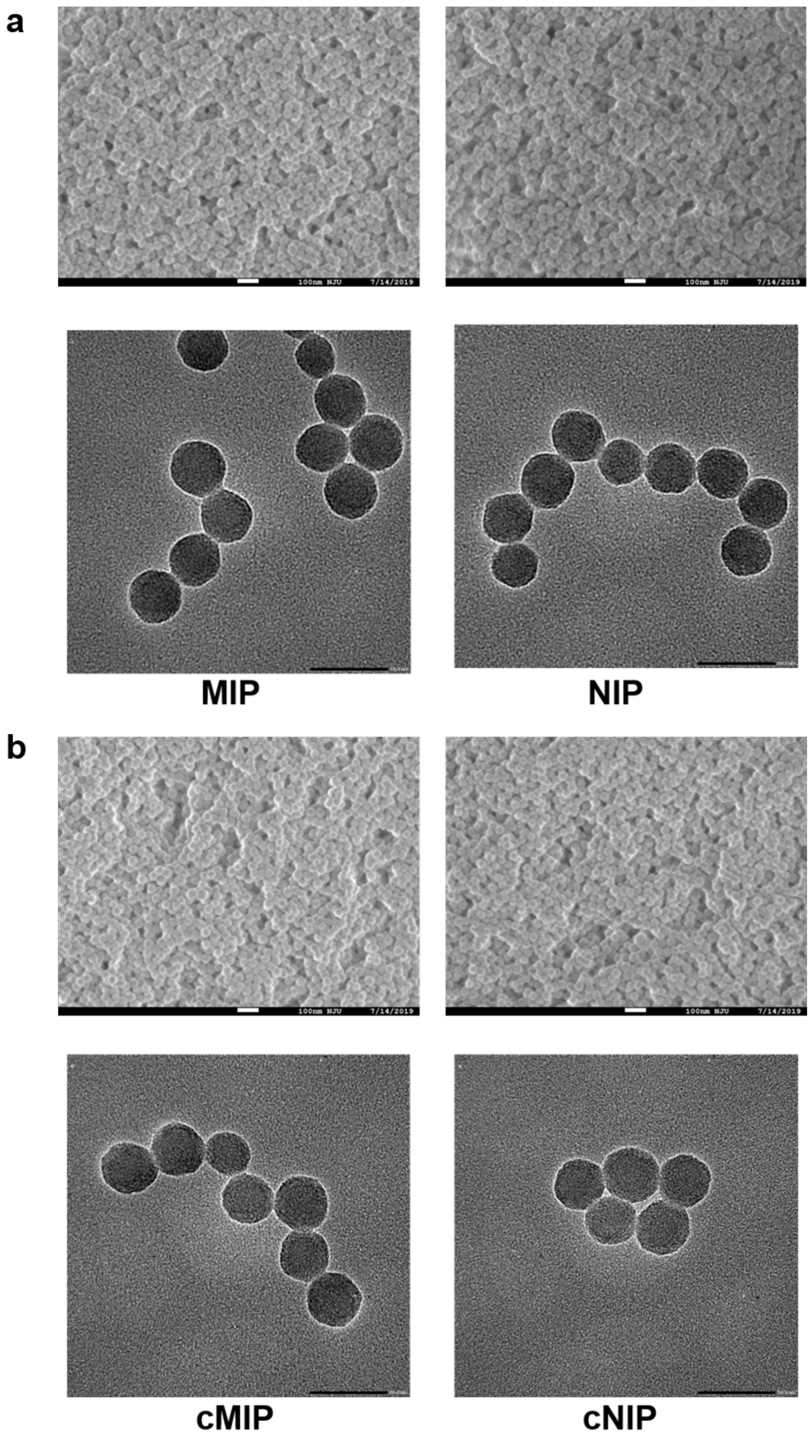

Figure S5. SEM and TEM characterization of nanoparticles prepared at the ratio of total monomers/TEOS of 10:90. a) MIP and NIP. b) cMIP and cNIP. The APTES/UPTES/IBTES/BnTES ratio was 20:20:50:10.

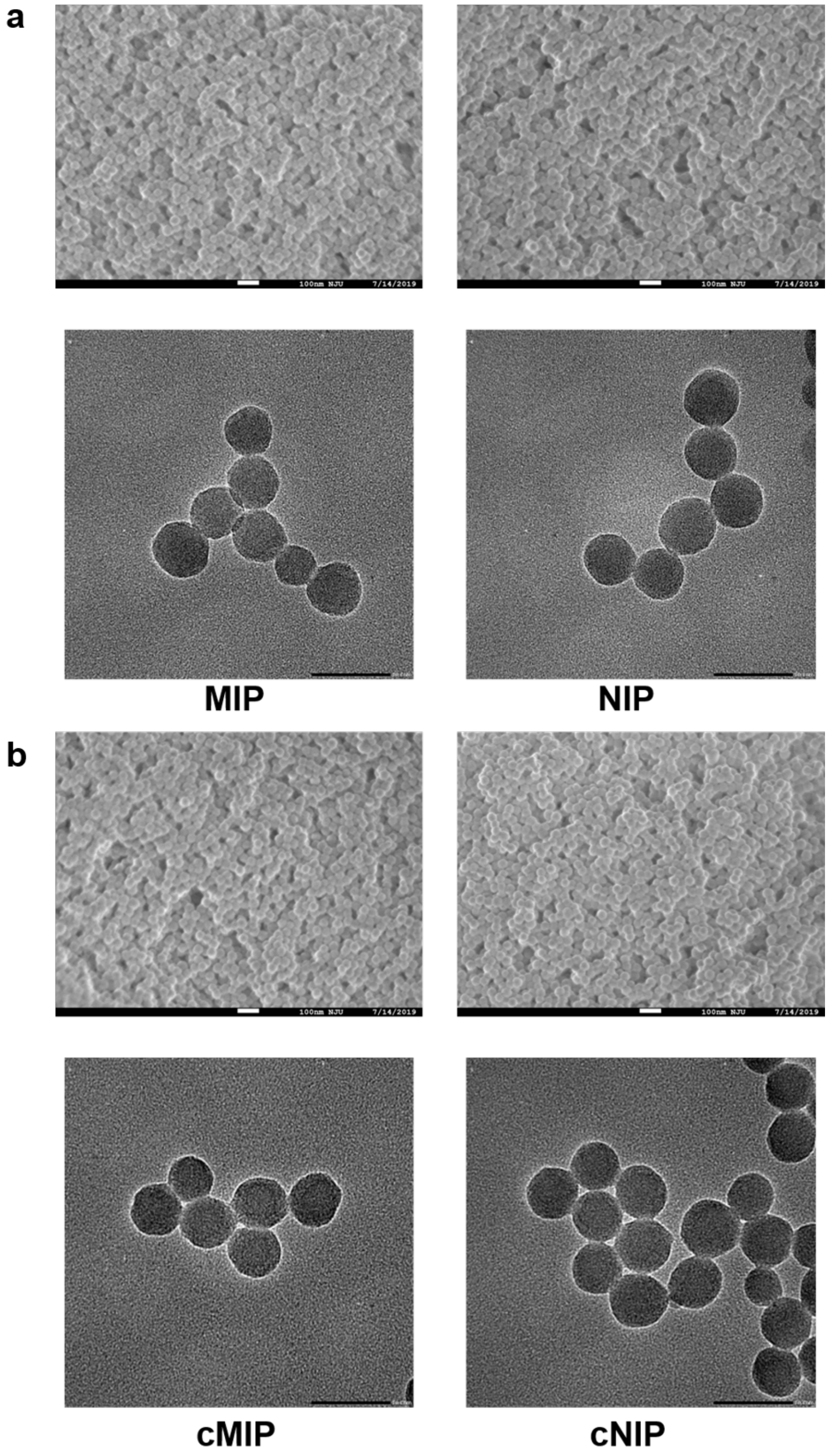

Figure S6. SEM and TEM characterization of nanoparticles prepared at the monomers/TEOS ratio of 15:85. a) MIP and NIP. b) cMIP and cNIP. The ratio of APTES/UPTES/IBTES/BnTES was fixed at 20:20:50:10.

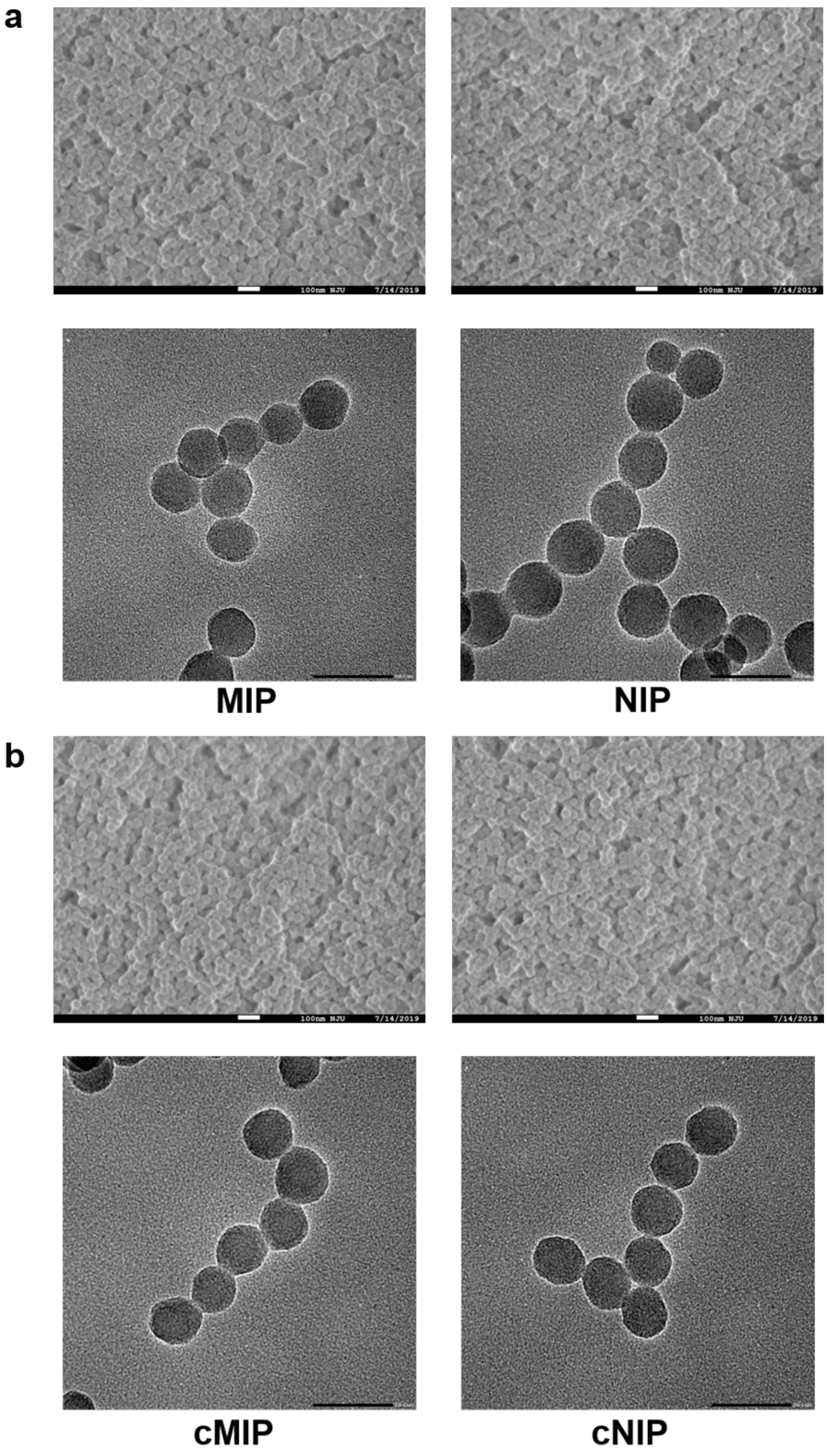

Figure S7. SEM and TEM characterization of nanoparticles prepared at the total monomers/TEOS ratio of 20:80. a) MIP and NIP. b) cMIP and cNIP. The APTES/UPTES/IBTES/BnTES ratio was 20:20:50:10.

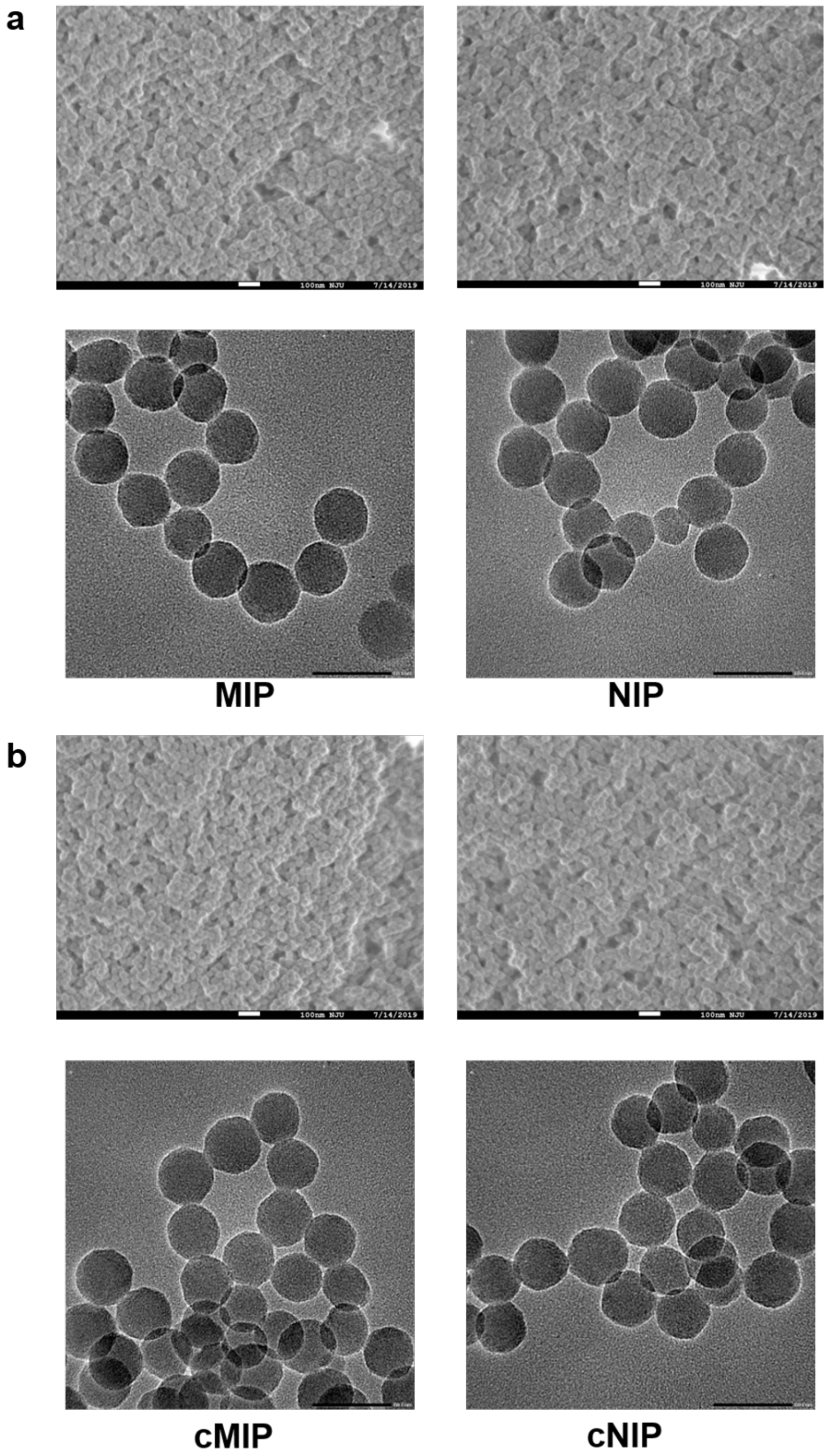

Figure S8. SEM and TEM characterization of nanoparticles prepared at the total monomers/TEOS ratio of 25:75. a) MIP and NIP. b) cMIP and cNIP. The APTES/UPTES/IBTES/BnTES ratio was 20:20:50:10.

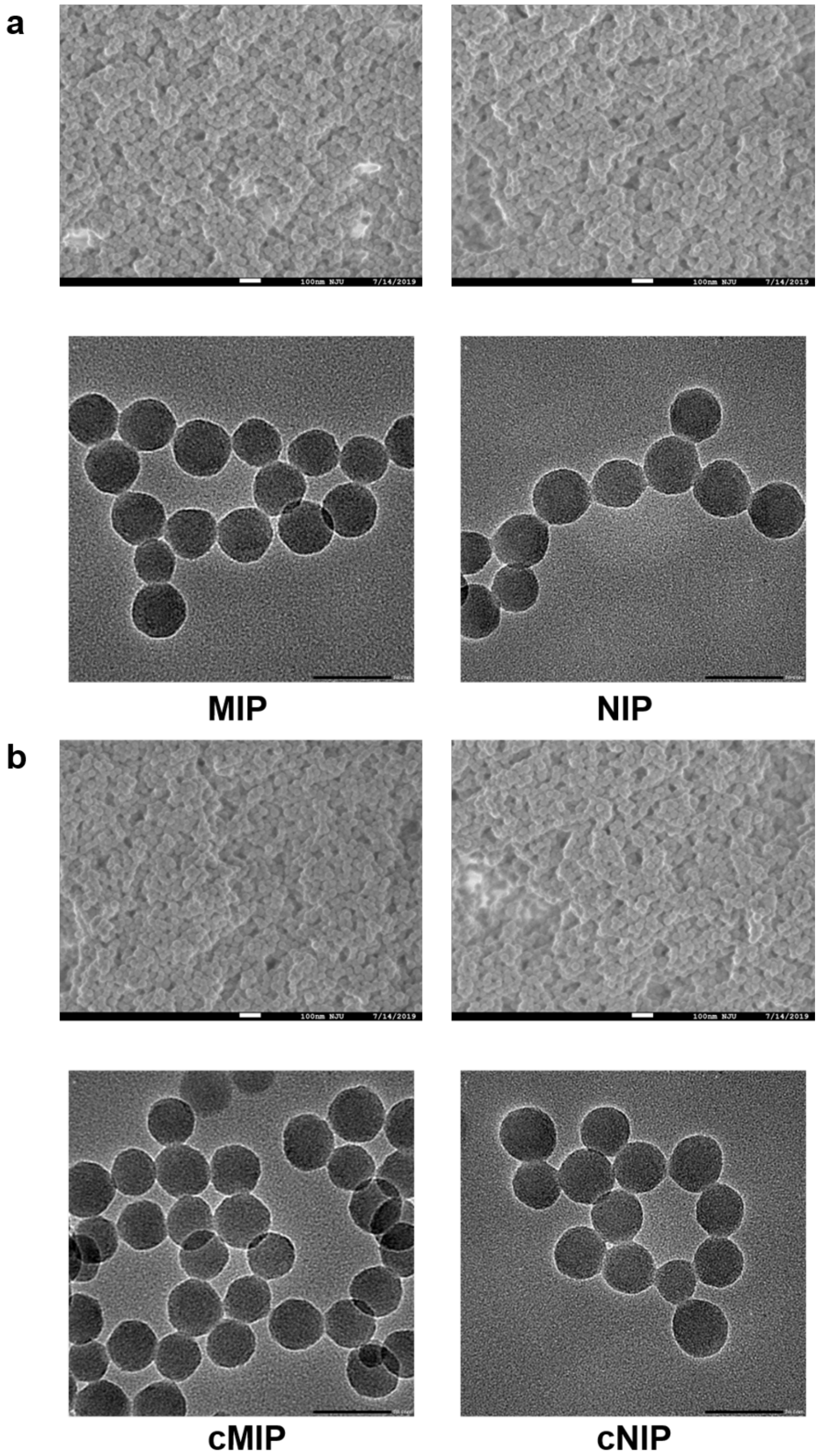

Figure S9. SEM and TEM characterization of nanoparticles prepared at the total monomer/TEOS ratio of 30:70. a) MIP and NIP. b) cMIP and cNIP. The APTES/UPTES/IBTES/BnTES ratio was 20:20:50:10.

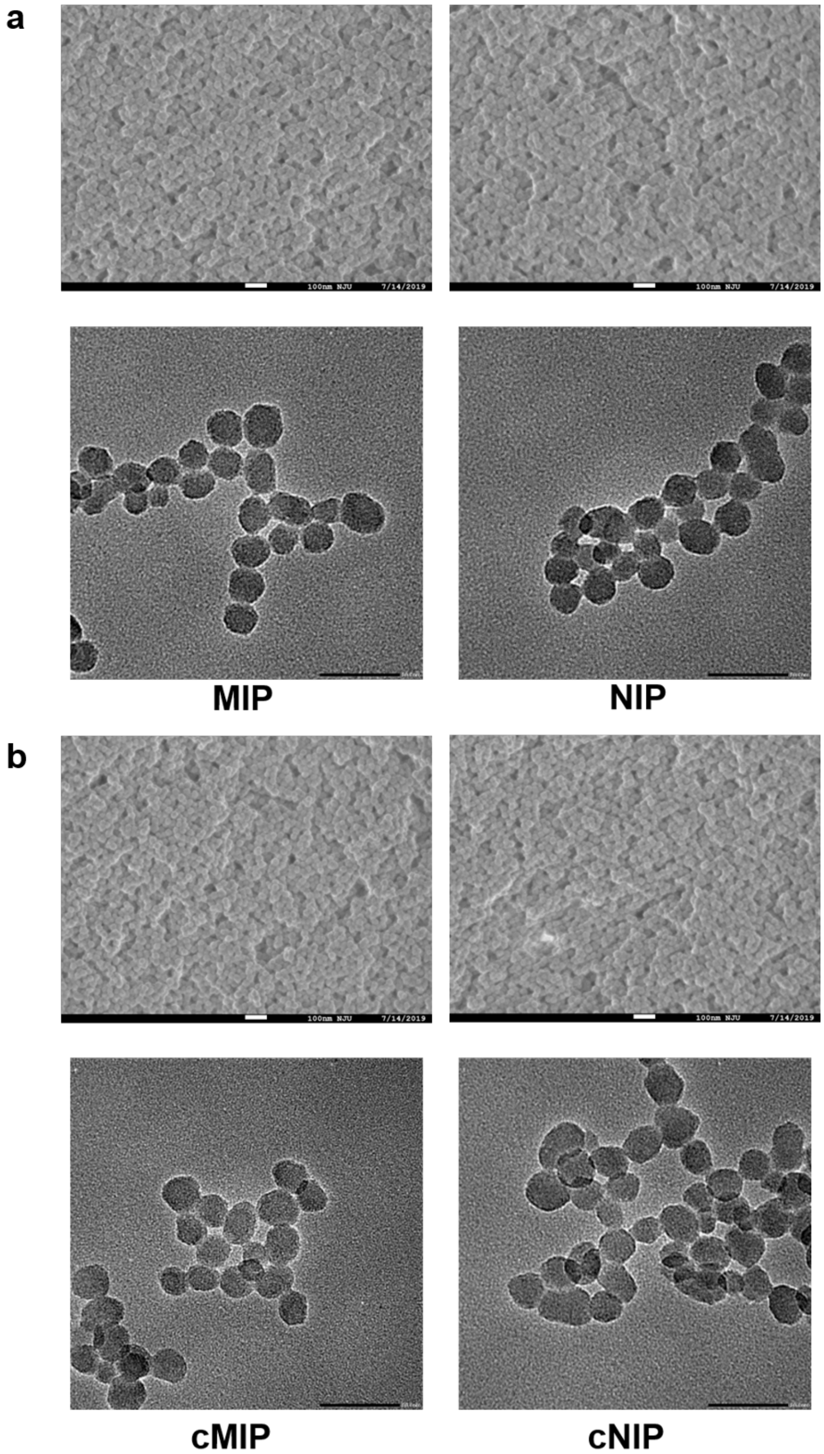

Figure S10. SEM and TEM characterization of nanoparticles prepared at the total monomer/TEOS ratio of 40:60. a) MIP and NIP. b) cMIP and cNIP. The APTES/UPTES/IBTES/BnTES ratio was 20:20:50:10.

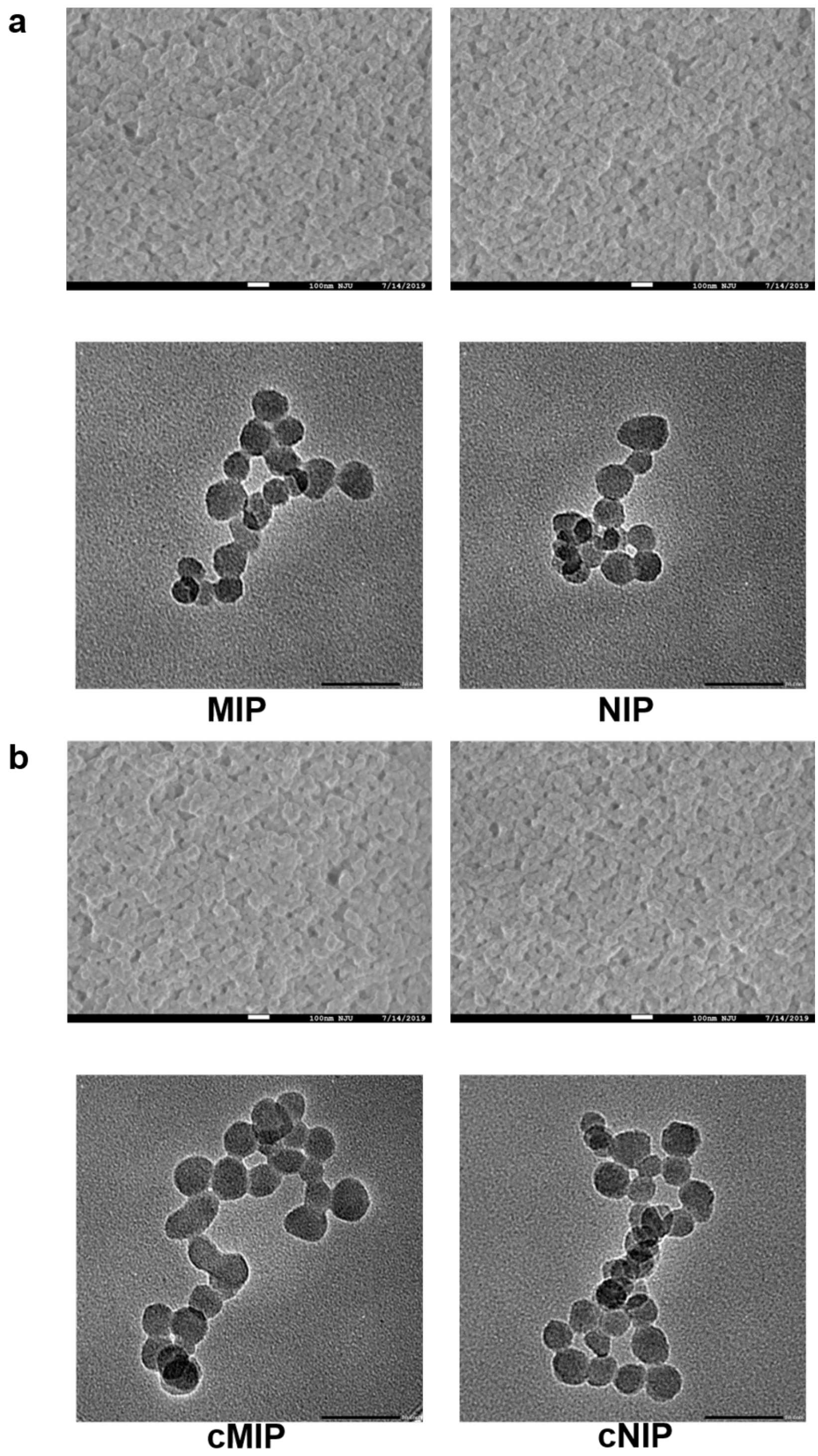

Figure S11. SEM and TEM characterization of nanoparticles prepared at the total monomer/TEOS ratio of 50:50. a) MIP and NIP. b) cMIP and cNIP. The APTES/UPTES/IBTES/BnTES ratio was 20:20:50:10.

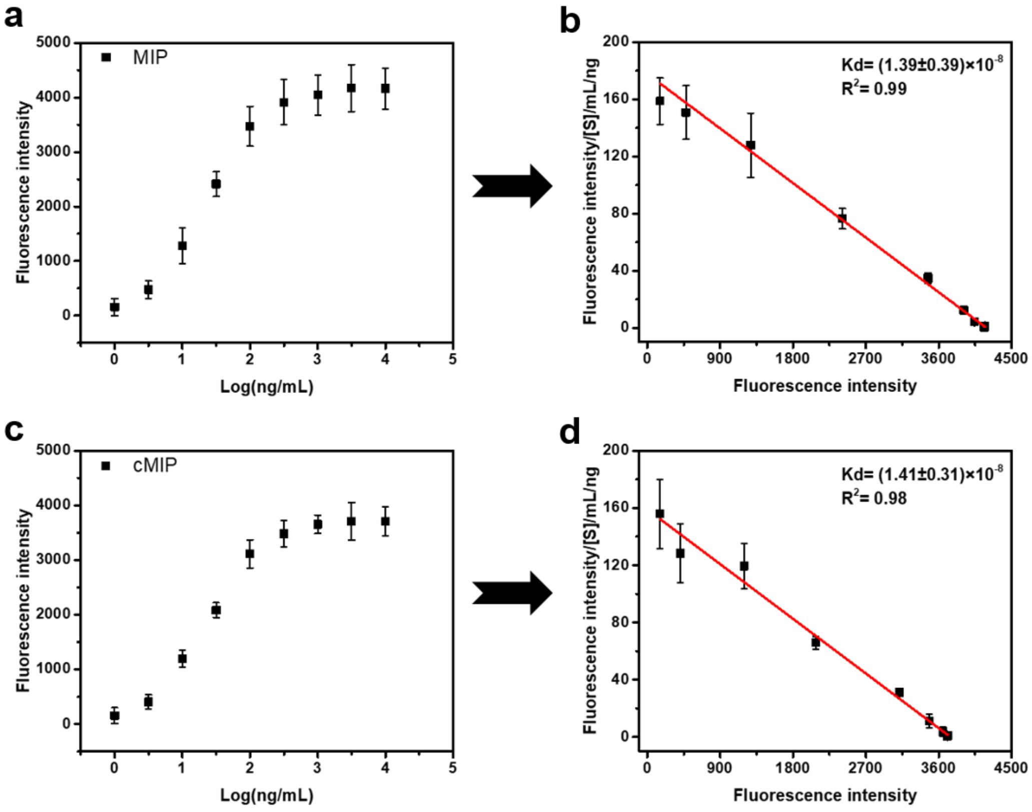

Figure S12. Binding isotherms and affinity measurement. Binding isotherm (a) and Scatchard plot (b) for the binding of B2M C-terminal epitope-imprinted MIP prepared at the monomer ratio of APTES/UPTES/BnTES/IBTES = 20:20:50:10 and the ratio of monomers/TEOS = 20:80. Binding isotherm (c) and Scatchard plot (d) for the binding of B2M C-terminal epitope-imprinted cMIP prepared at the monomer ratio of APTES/UPTES/BnTES/IBTES = 20:20:50:10 and the ratio of monomers/TEOS = 30:70.

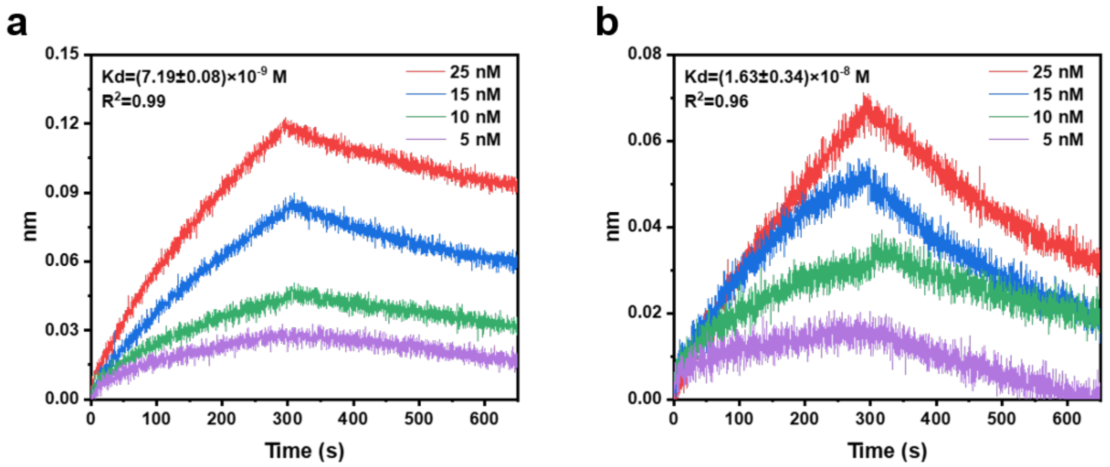

Figure S13. Binding affinity measurement of MIP and cMIP towards the intact B2M protein by BLI. a) The BLI binding curves of B2M C-terminal epitope-imprinted MIP prepared at the monomer ratio of APTES/UPTES/BnTES/IBTES = 20:20:50:10 and the ratio of monomers/TEOS = 20:80. b) The BLI binding curves of B2M C-terminal epitope-imprinted cMIP prepared at the monomer ratio of APTES/UPTES/BnTES/IBTES = 20:20:50:10 and the ratio of monomers/TEOS = 30:70.

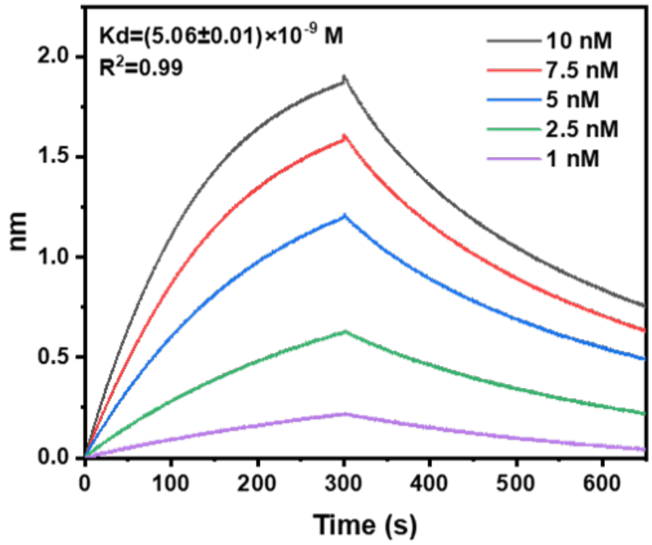

Figure S14. Binding affinity measurement of anti-B2M monoclonal antibody by BLI. The BLI binding curves of anti-B2M antibody towards the intact B2M protein.

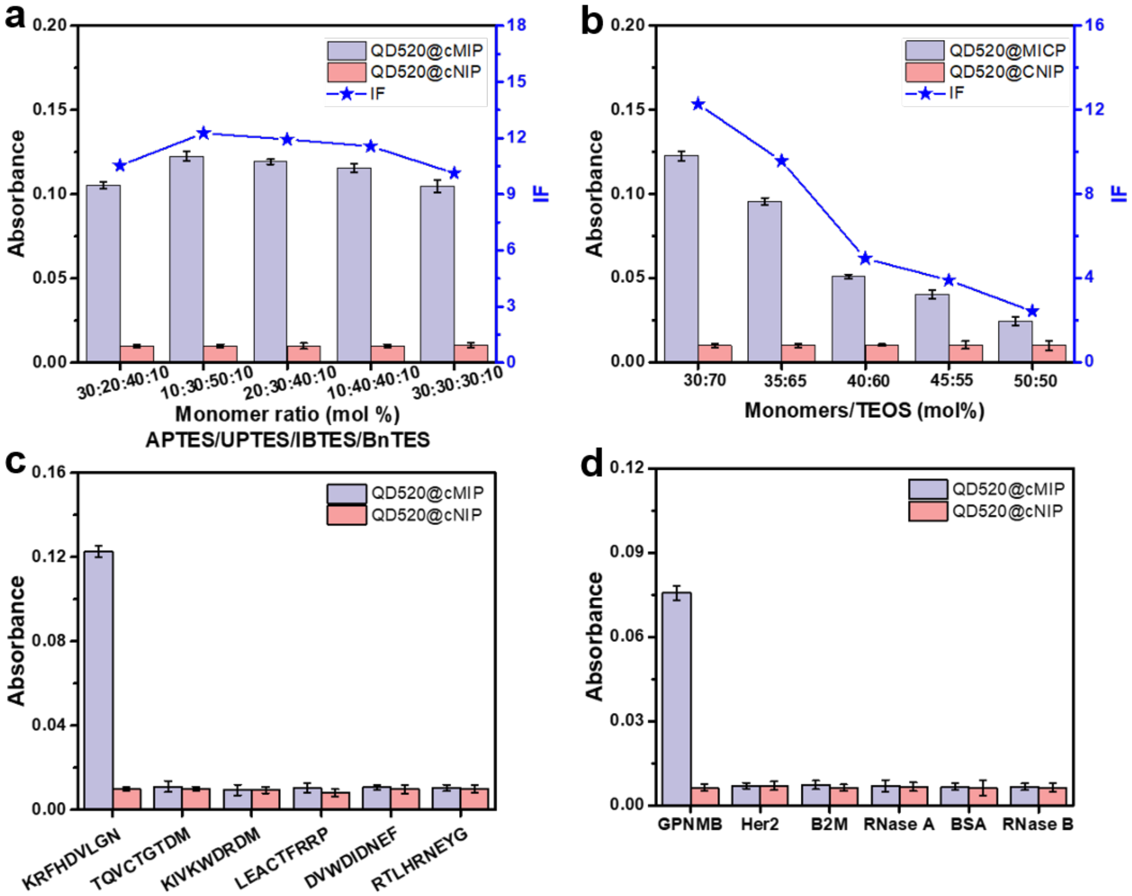

Figure S15. Optimization and selectivity test of GPNMB N-terminal epitope-imprinted QD520@cMIP. Optimization of the monomer ratio of (a) and the total monomers/TEOS ratio (b), and the selectivity test of QD520@cMIP toward different peptides (c) and proteins (d).

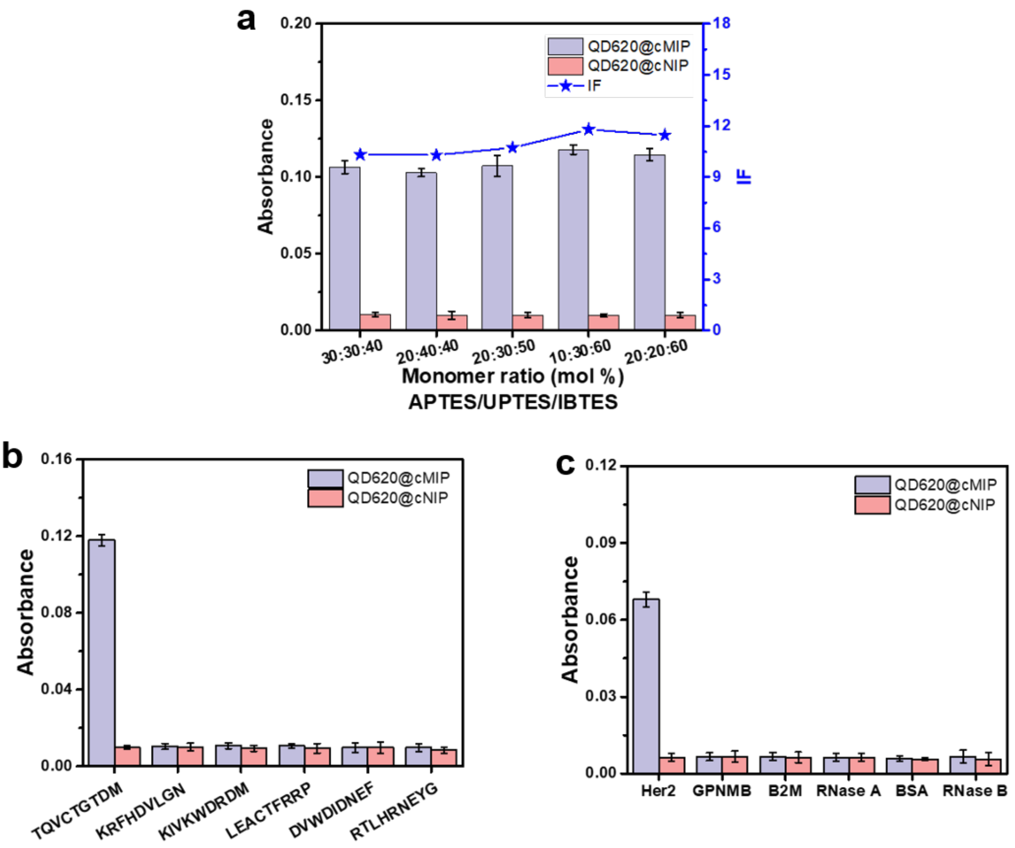

Figure S16. Optimization and selectivity test of HER2 N-terminal epitope-imprinted QD620@cMIP. Optimization of the monomer ratio of (a) and the selectivity test of QD620@cMIP towards different peptides (b) and proteins (c).

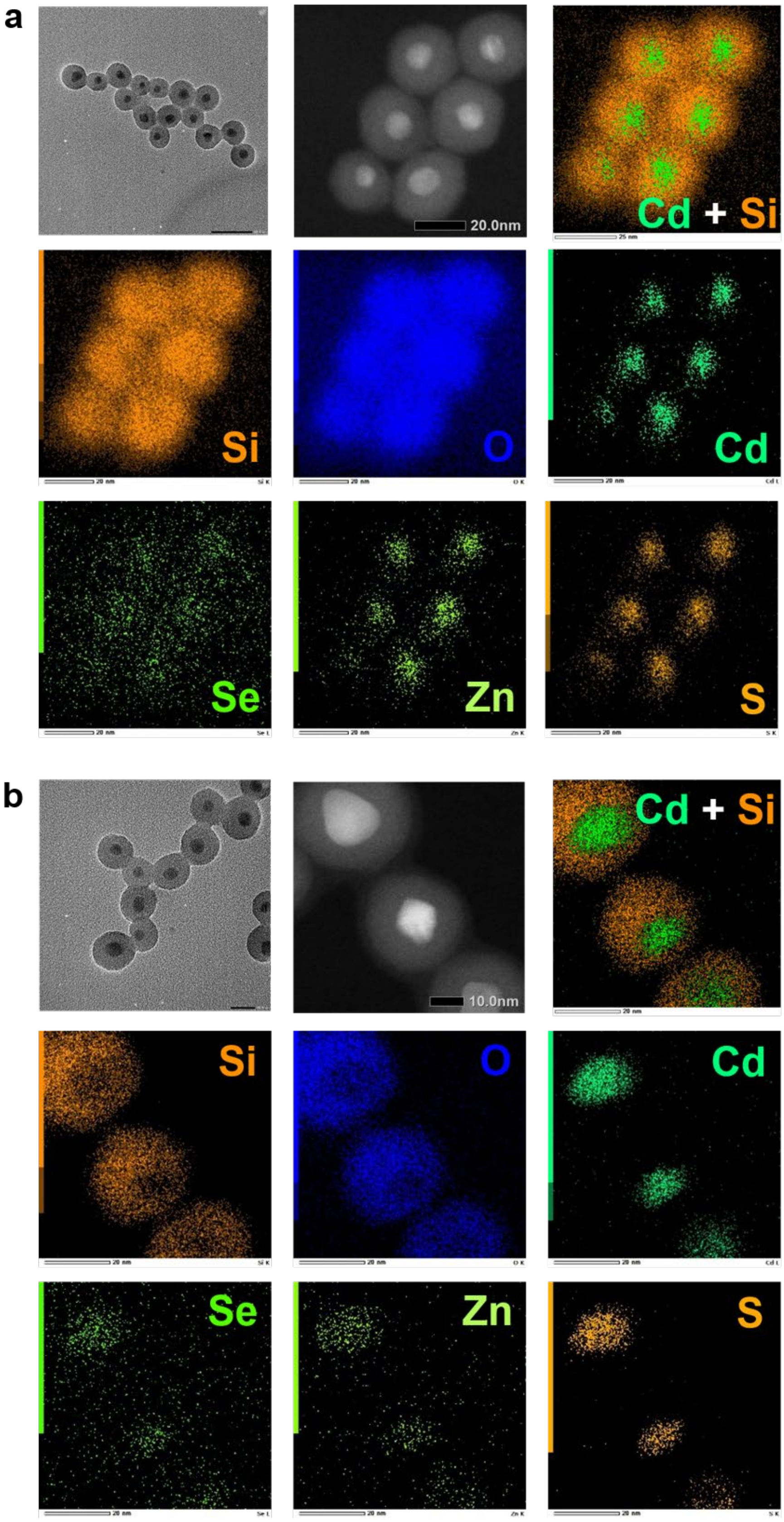

Figure S17. TEM, STEM and EDS mapping of GPNMB-specific QD520@cMIP (a) and HER2-specific QD620@cMIP (b).

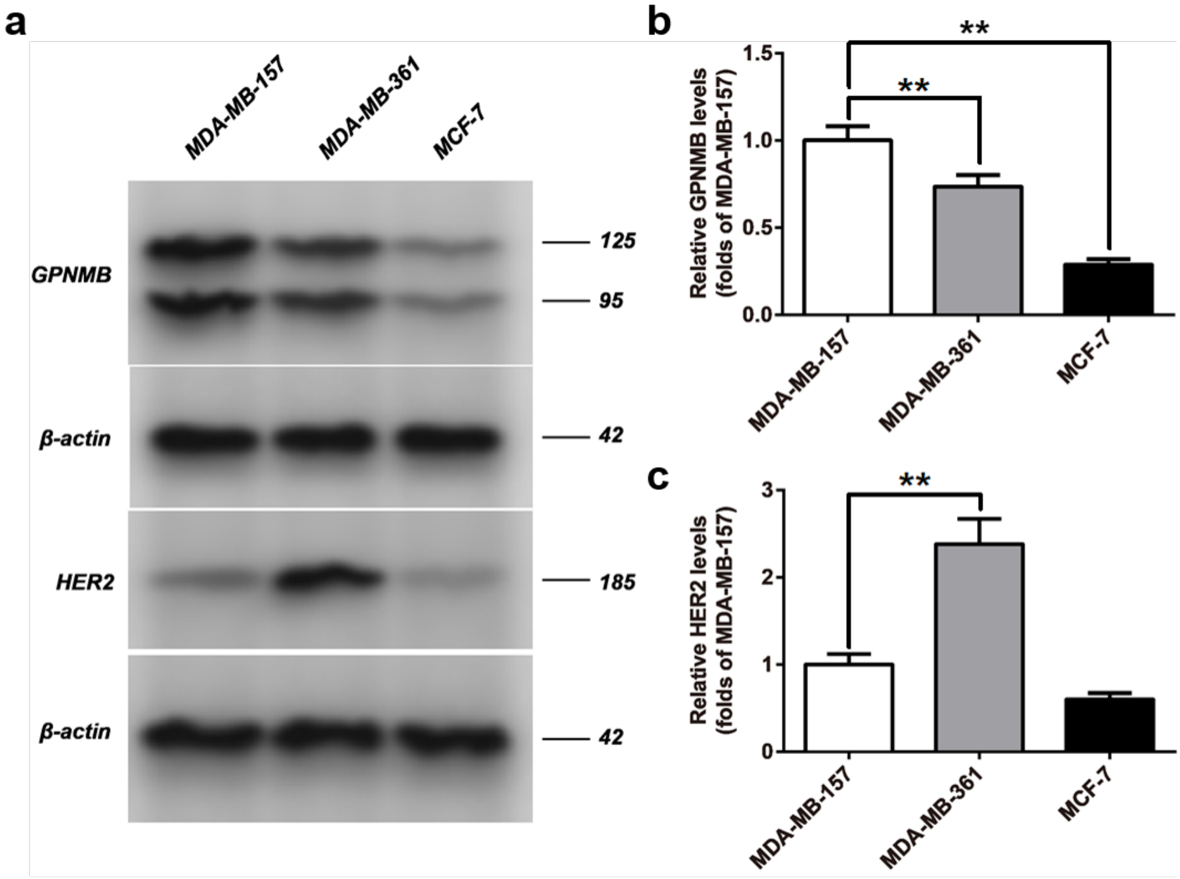

Figure S18. Western blot assay of the expression of GPNMB and HER2 from different cell lines (a) and the normalized levels of GPNMB (b) and HER2 (c) in different cell lines relative to MDA-MB-157. The results are presented as mean ± SD (n = 3). *p < 0.05, **p < 0.01 vs. MDA-MB-157 group.

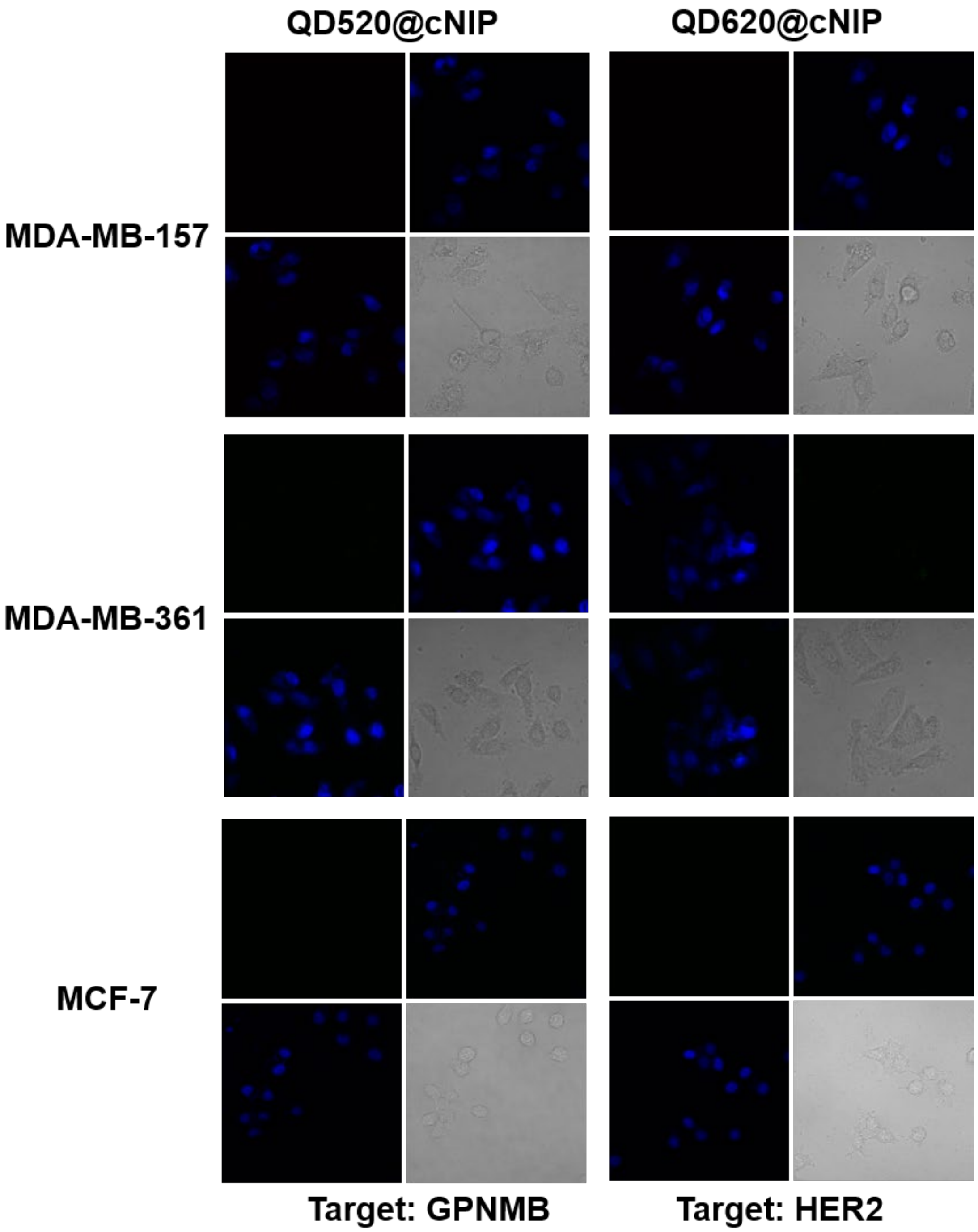

Figure S19. Confocal fluorescence imaging of MDA-MB-157, MDA-MB-361 and MCF-7 cells after staining with QD520@cNIP or QD620@cNIP. Blue: nuclei stained by Hoechst 33342.

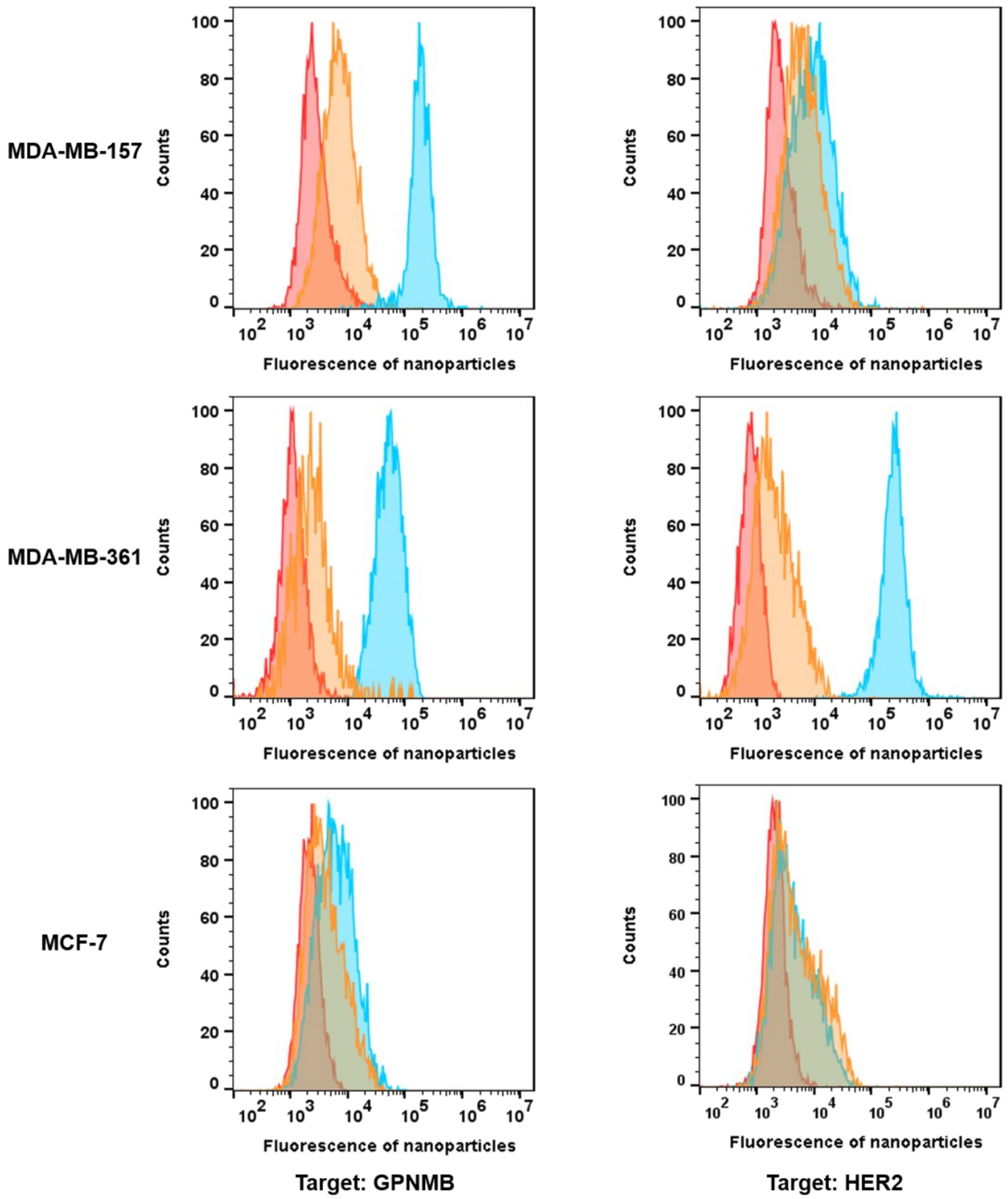

Figure S20. Quantitative flow cytometry of MDA-MB-157, MDA-MB-361 and MCF-7 cells after staining with GPNMB-specific QD520@cMIP, QD520@cNIP, HER2-specific QD620@cMIP or QD620@cNIP. Blue: staining by GPNMB-specific QD520@cMIP for GPNMB as the target and HER2-specific QD620@cMIP for HER2 as the target. Orange: staining by QD520@cNIP for GPNMB as the target and QD620@cNIP for HER2 as the target. Red: without staining (control).

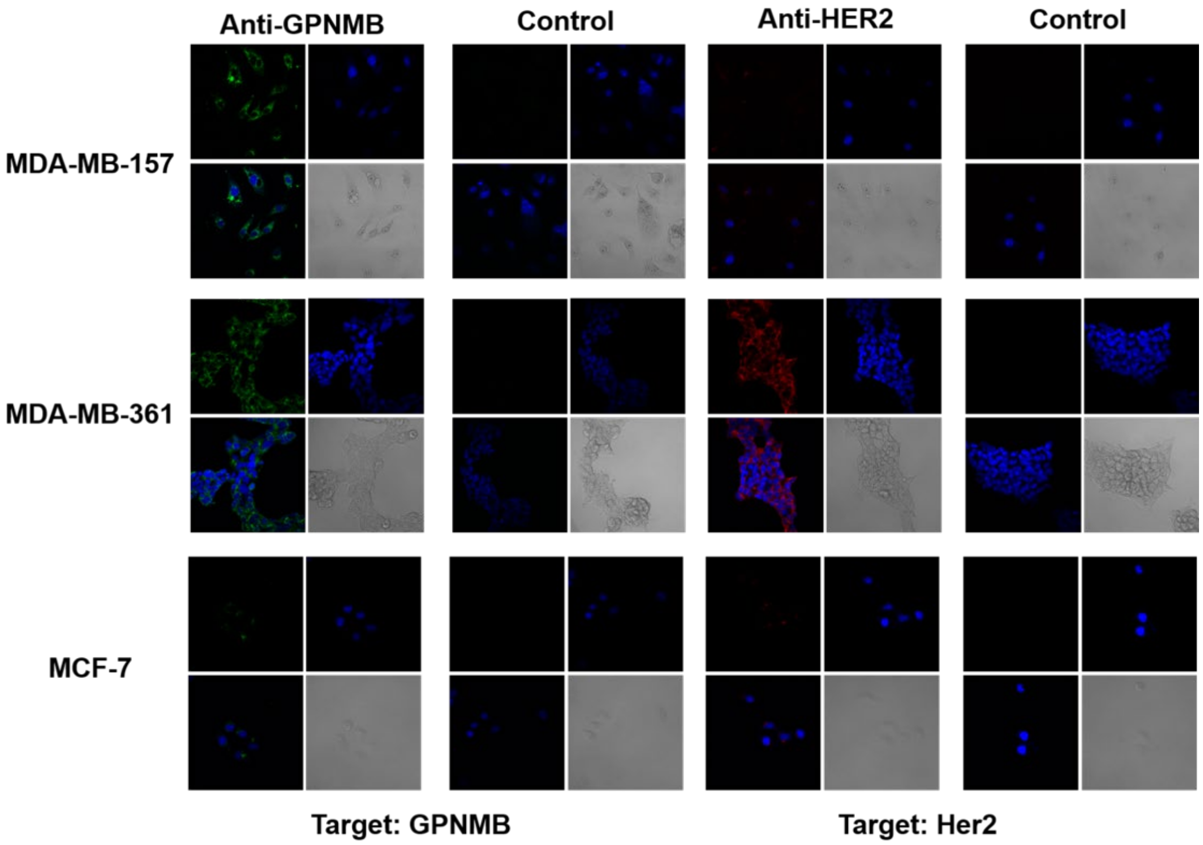

Figure S21. Immunofluorescent imaging of MDA-MB-157, MDA-MB-361 and MCF-7 cells after staining with anti-GPNMB antibody or anti-HER2 antibody followed by fluorescent goat anti-rabbit IgG H&L secondary antibody. Blue: nuclei stained by DAPI. Green: staining by FITC-conjugated goat anti-rabbit IgG H&L secondary antibody. Red: staining by Alexa Fluor® 647-conjugated goat anti-rabbit IgG H&L secondary antibody.

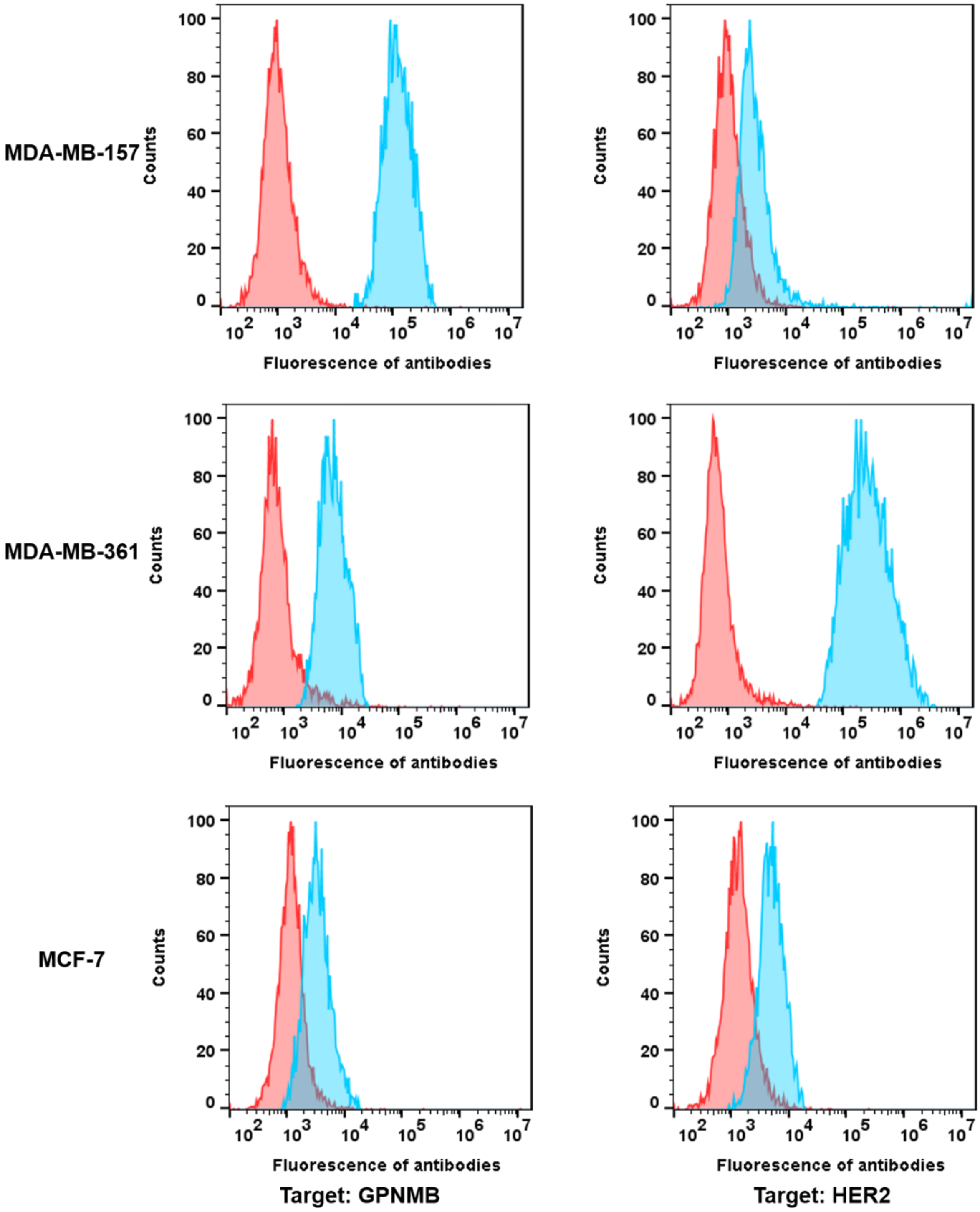

Figure S22. Quantitative flow cytometry of MDA-MB-157, MDA-MB-361 and MCF-7 cells after staining with anti-GPNMB antibody or anti-HER2 antibody followed by FITC-conjugated goat anti-rabbit IgG H&L secondary antibody. Blue: staining by anti-GPNMB antibody or anti-HER2 antibody followed by FITC-conjugated goat anti-rabbit IgG H&L secondary antibody. Red: without staining (control).

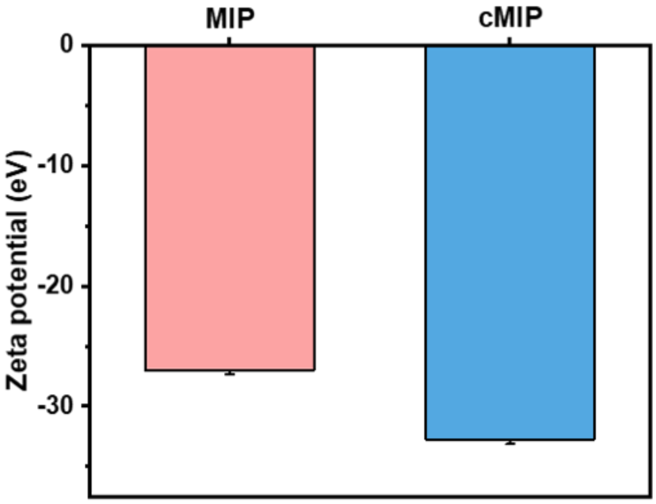

Figure S23. Zeta potential of MIP and cMIP in water. The MIP used was B2M C-terminal epitope-imprinted MIP prepared at the monomer ratio of APTES/UPTES/BnTES/IBTES = 20:20:50:10 and the ratio of monomers/TEOS = 20:80. The cMIP used was B2M C-terminal epitope-imprinted cMIP prepared at the monomer ratio of APTES/UPTES/BnTES/IBTES = 20:20:50:10 and the ratio of monomers/TEOS = 30:70.

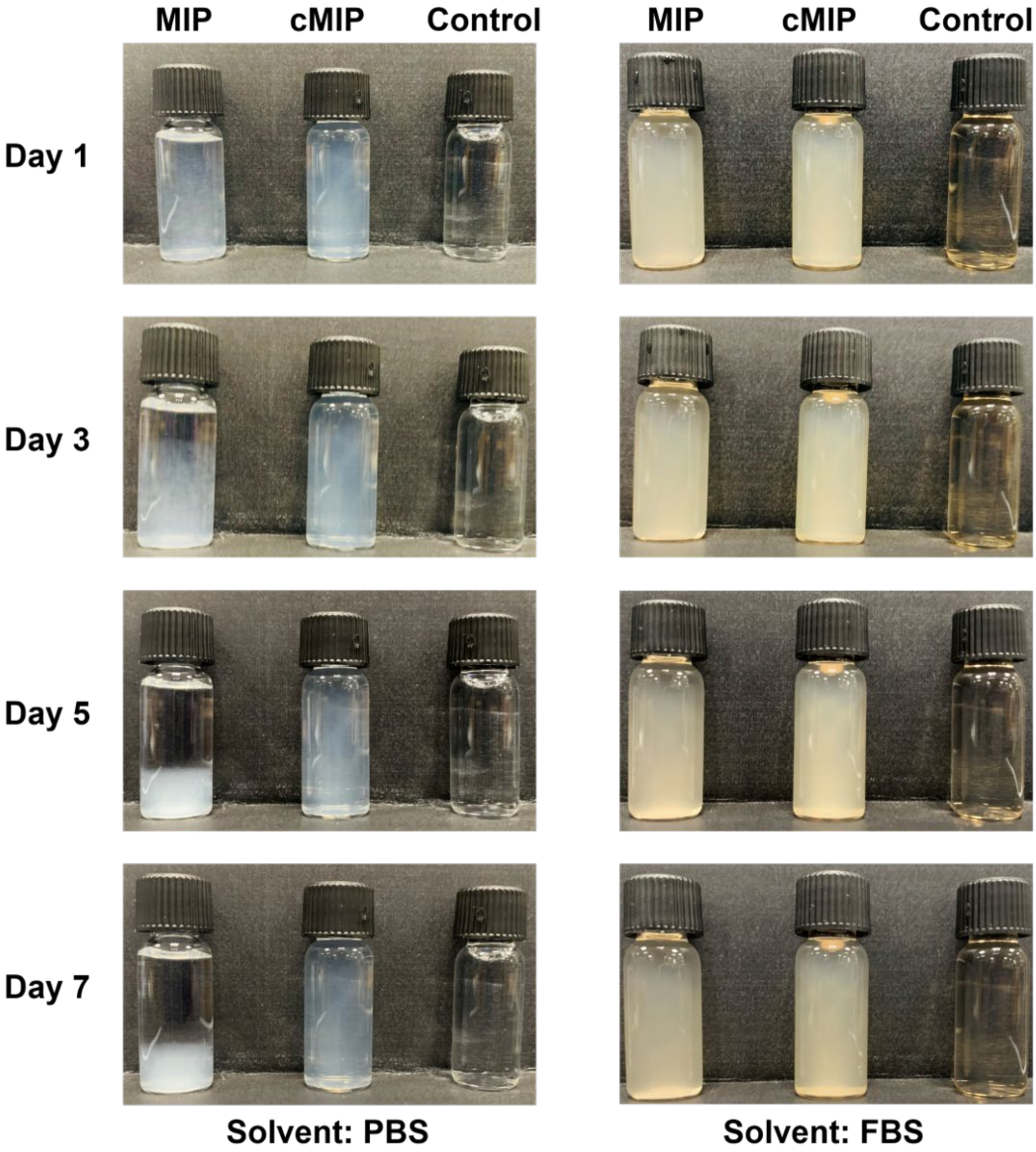

Figure S24. In vitro stability evaluation of MIP and cMIP in PBS and fetal bovine serum (FBS). The MIP used was B2M C-terminal epitope-imprinted MIP prepared at the monomer ratio of APTES/UPTES/BnTES/IBTES = 20:20:50:10 and the ratio of monomers/TEOS = 20:80. The cMIP used was B2M C-terminal epitope-imprinted cMIP prepared at the monomer ratio of APTES/UPTES/BnTES/IBTES = 20:20:50:10 and the ratio of monomers/TEOS = 30:70. Control: PBS or FBS without the presence of MIP or cMIP.

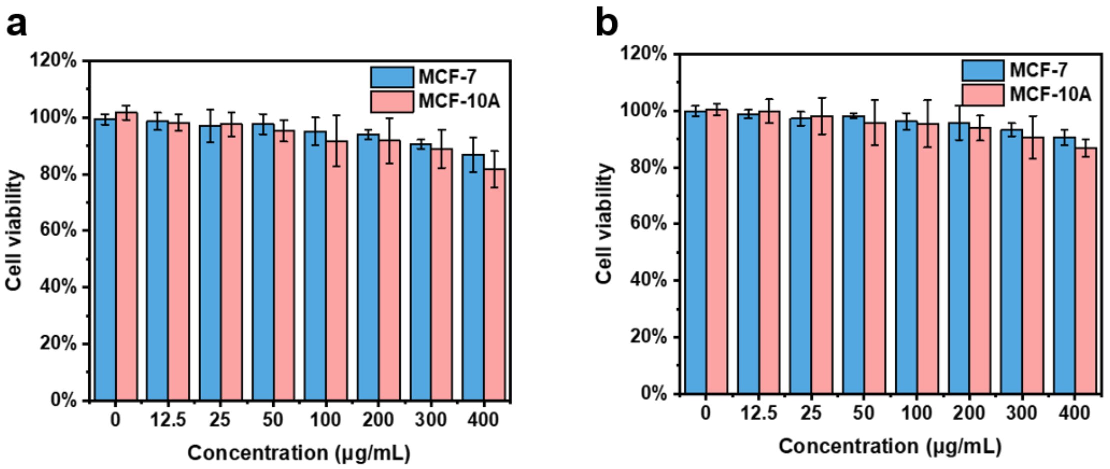

Figure S25. Cell viability of MCF-7 cells and MCF-10A cells treated with different concentrations of anti-GPNMB QD520@MIP (a) and anti-GPNMB QD520@cMIP (b).

Table S1. Binding parameters for anti-B2M monoclonal antibody, B2M C-terminal epitope-imprinted MIP and cMIP to the intact protein B2M by BLI.

Item	K_d / M	R^2	k_{on} / M^{-1} s^{-1}	k_{off} / s^{-1}
Antibody	5.06×10^{-9}	0.99	5.53×10^{5}	2.80×10^{-3}
MIP	7.19×10^{-9}	0.99	1.19×10^{5}	8.55×10^{-4}
cMIP	1.63×10^{-8}	0.96	7.08×10^{4}	1.15×10^{-3}
